# Exact Exploratory Bi-factor Analysis: A Constraint-Based Optimization Approach

**DOI:** 10.1017/psy.2025.17

**Published:** 2025-05-16

**Authors:** Jiawei Qiao, Yunxiao Chen, Zhiliang Ying

**Affiliations:** 1 School of Mathematical Sciences, Fudan University, Shanghai, China; 2 Department of Statistics, London School of Economics and Political Science, London, UK; 3 Department of Statistics, Columbia University, New York, NY, USA

**Keywords:** augmented Lagrangian method, bi-factor model, exploratory bi-factor analysis, hierarchical factor model

## Abstract

Bi-factor analysis is a form of confirmatory factor analysis widely used in psychological and educational measurement. The use of a bi-factor model requires specifying an explicit bi-factor structure on the relationship between the observed variables and the group factors. In practice, the bi-factor structure is sometimes unknown, in which case, an exploratory form of bi-factor analysis is needed. Unfortunately, there are few methods for exploratory bi-factor analysis, with the exception of a rotation-based method proposed in Jennrich and Bentler ([2011, Psychometrika 76, pp. 537–549], [2012, Psychometrika 77, pp. 442–454]). However, the rotation method does not yield an exact bi-factor loading structure, even after hard thresholding. In this article, we propose a constraint-based optimization method that learns an exact bi-factor loading structure from data, overcoming the issue with the rotation-based method. The key to the proposed method is a mathematical characterization of the bi-factor loading structure as a set of equality constraints, which allows us to formulate the exploratory bi-factor analysis problem as a constrained optimization problem in a continuous domain and solve the optimization problem with an augmented Lagrangian method. The power of the proposed method is shown via simulation studies and a real data example.

## 1 Introduction

The bi-factor model was originally proposed by Holzinger and Swineford (1937) for linear factor analysis and further extended by Gibbons and Hedeker (1992), Gibbons et al. (2007), Cai et al. (2011), among others, to nonlinear factor analysis settings to account for dichotomous, ordinal, and nominal data. These models assume that the observed variables can be accounted for by 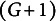 factors, with a general factor, onto which all items load directly, and *G* group factors that each is associated with a subset of variables. Such a specification leads to good interpretations in many real-world applications. These models have received wide applications in psychological and educational measurement (see, e.g., Bradlow et al., 1999; Cai et al., 2016; Chen et al., 2012; DeMars, 2006, 2012; Gibbons et al., 2009; Gignac & Watkins, 2013; Jeon et al., 2013; Reise et al., 2007; Rijmen, 2010). However, we note that all these applications of bi-factor analysis are confirmatory in the sense that one needs to pre-specify the number of group factors and the relationship between the observed variables and the group factors. Such prior knowledge may not always be available. In that case, an exploratory form of bi-factor analysis is needed.

Exploratory bi-factor analysis can be seen as a special case of exploratory factor analysis, which dates back to the seminal work of Thurstone (1947) concerning finding a “simple structure” of loadings. Various rotation methods have been proposed for exploratory factor analysis. A short list of relevant works includes Kaiser (1958), McCammon (1966), Jennrich and Sampson (1966), McKeon (1968), Crawford and Ferguson (1970), Yates (1987), Jennrich (2006), Jennrich (2004), Jennrich and Bentler (2011, 2012), and Liu et al. (2023). We refer the readers to Browne (2001) for a review of rotation methods for exploratory factor analysis.

However, standard exploratory factor analysis methods do not apply to the bi-factor analysis setting, and few methods have been developed for exploratory bi-factor analysis. An exception is the seminal work of Jennrich and Bentler (2011, 2012), who proposed a rotation-based method for exploratory bi-factor analysis with orthogonal or oblique factors. However, their approach has some limitations. First, as a common issue with rotation-based methods, their method does not yield many zero loadings, and thus, the resulting loading structure does not have an exact bi-factor structure. Although a post-hoc thresholding procedure (i.e., treating loadings with an absolute value below a threshold as zero) can be applied to obtain a cleaner loading pattern, it does not work well when some variables show relatively large loadings on more than one group factor after the rotation. In fact, one cannot always find a threshold that yields an exact bi-factor structure that each variable loads on one and only one group factor. Second, as noted in Jennrich and Bentler (2012), their method fails completely in the best case where there is a rotation of an initial loading matrix that has an exact bi-factor structure. This failure is due to that their rotation method cannot incorporate the zero constraints on the correlations between the general factor and the group factors.

This article proposes a constrained optimization method for exploratory bi-factor analysis, which overcomes the issues with the rotation-based method. The contribution is four-fold. First, we provide a mathematical characterization of the bi-factor loading structure as a set of nonlinear equality constraints, which allows us to formulate the exploratory bi-factor analysis problem as a constrained optimization problem. In other words, it turns a discrete model selection problem into a continuous optimization problem, which reduces the computational demand in some sense. It is shown that in the aforementioned best case where the rotation method fails, the global solutions to the optimization can perfectly recover the true bi-factor structure. Second, we propose an augmented Lagrangian method (ALM; Bertsekas, 2014) for solving this optimization problem, which is a standard numerical optimization method for solving constrained optimization with robust empirical performance and good theoretical properties. Third, we combine the proposed method with the Bayesian information criterion (BIC; Schwarz, 1978) for selecting the number of group factors. Compared with existing exploratory factor analysis methods for determining the number of factors, our method is tailored to the bi-factor model structure and, thus, tends to be statistically more efficient when the data is indeed generated by a bi-factor model. Finally, we demonstrate that the proposed method can be extended to learning the loading structure of hierarchical factor models (Schmid & Leiman, 1957; Yung et al., 1999), a higher-order extension of the bi-factor model that has received wide applications (see, e.g., Brunner et al., 2012, and the references therein). The bi-factor model can be viewed as a special hierarchical factor model with a two-layer factor structure, with the general factor in one layer and the group factors in the other. Similar to exploratory bi-factor analysis, the proposed method yields exact hierarchical factor loading structures without a need for post-hoc treatments.

The rest of the article is organized as follows. In Section 2, we formulate the exploratory bi-factor analysis problem as a constrained optimization problem and propose an ALM for solving it. We also propose a BIC-based procedure for selecting the number of group factors. Simulation studies and a real data example are presented in Sections 3 and 4, respectively, to evaluate the performance of the proposed method. We conclude with discussions in Section 5. The Appendix in the Supplementary Material includes additional details about the computation, the simulation studies and the real data example, an extension of the proposed method to exploratory hierarchical factor analysis, and proof of the theoretical results.

## 2 Exploratory bi-factor analysis by constrained optimization

### 2.1 Bi-factor model structure and a constrained optimization formulation

For the ease of exposition and simplification of the notation, we focus on the linear bi-factor model, while noting that the constraints that we derive for the bi-factor loading matrix below can be combined with the likelihood function of other bi-factor models (e.g., Cai et al., 2011; Gibbons et al., 2007; Gibbons & Hedeker, 1992) for their exploratory analysis. We focus on the extended bi-factor model, also known as the oblique bi-factor model, as considered in Jennrich and Bentler (2012) and Fang et al. (2021). This model is more general than the standard bi-factor model, in the sense that the latter assumes all the factors to be uncorrelated while the former allows the group factors to be correlated. As established in Fang et al. (2021), this extended bi-factor model is identifiable under mild conditions. We also point out that the proposed method can be easily adapted to the standard bi-factor model.

Consider a dataset with *N* observation units from a certain population and *J* observed variables. The extended bi-factor model assumes that there exists a general factor and *G* group factors. The group factors are loaded by independent clusters of variables, where each variable belongs to only one cluster. The model further assumes that the population covariance matrix of the observed variables can be decomposed as 

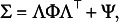

where 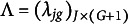 is the loading matrix, 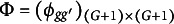 is the correlation matrix of the factors, which is assumed to be strictly positive definite, and 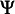 is a 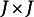 diagonal matrix with diagonal entries 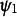 , …, 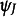 . Let 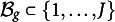 denote the cluster of variables loading on the *g*th group factor. Then the bi-factor model assumption implies that 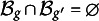 , 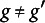 , and 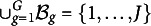 . The following zero constraints on the loading matrix 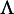 hold: 

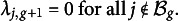

In addition, the correlation matrix 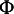 satisfies 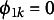 for all 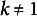 , meaning that all the group factors are uncorrelated with the general factor. This constraint on 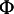 is necessary to ensure that the extended bi-factor model is identifiable (Fang et al., 2021), as otherwise, there will be a rotational indeterminacy issue.

Now suppose that the number of group factors *G* is known, while the clusters 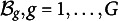 , are unknown. Section 2.3 considers the selection of *G* when it is unknown. The bi-factor structure means that for each *j*, there is at most one nonzero element in 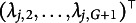 . Consequently, the loading matrix 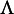 should satisfy the following 
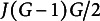
 constraints: 
(1)

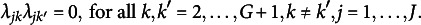



Therefore, the exploratory bi-factor analysis problem can be translated into the following constrained optimization problem: 
(2)

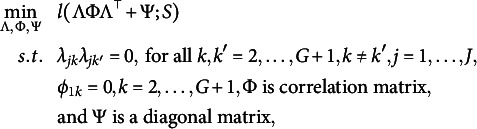

where *l* is a loss function and *S* is the sample covariance matrix of observed data. We focus on the case when *l* is the fit function based on the normal likelihood 

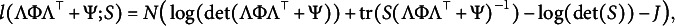

while noting that this loss function can be replaced by other loss functions for factor analysis (see, e.g., Chen et al., 2023), including the Frobenius norm of 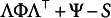 that is used in the least square estimator for factor analysis. We can also replace the sample covariance matrix in (2) with the sample correlation matrix, which is equivalent to performing exploratory bi-factor analysis based on variance-standardized variables.

The following theorem shows that the proposed method can perfectly recover the true bi-factor loading structure in the best case when 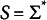 , where 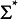 is the true covariance matrix of data. Note that the rotation method proposed in Jennrich and Bentler (2012) completely fails in this case. Before giving the statement of the theorem, we introduce some additional notations. For any matrix 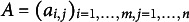 , 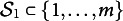 and 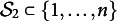 , let 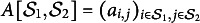 be the submatrix of *A* consisting of elements that lie in rows belonging to set 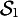 and columns belonging to set 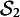 . For example, consider a matrix *A* with more than two rows and three columns. With index sets 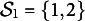 and 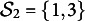 , the submatrix 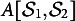 is a two-by-two matrix, taking the form 

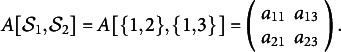

For any set 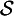 , let 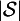 be the cardinality of 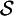 .

Let 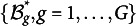 be the true nonoverlapping clusters of the *J* variables, satisfying for each 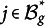 , 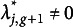 , 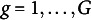 . Further, let 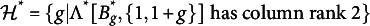 be the set of group factors for which the group loadings are linearly independent of the corresponding common loadings. Let 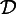 be the set of diagonal matrix with its diagonal entries taking values either 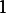 or 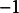 , and let 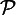 be the set of permutation matrix *P* such that each row and column of *P* has exactly one nonzero entry of value 1 and 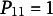 . Each matrix in 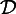 corresponds to a simultaneous sign flip of certain factors and the corresponding loading parameters. Each matrix in 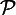 corresponds to a swapping of certain columns in the loading matrix associated with the group factors or, equivalently, a relabeling of the group factors. They are introduced to account for the sign-indeterminacy of the 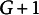 factors and the label indeterminacy of the group factors, respectively. See Theorem 1 and Remark 1 for more explanations.

Let 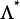 , 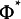 , and 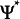 be the true values of the corresponding parameter matrices. The following conditions are sufficient for the identifiability of the bi-factor structure and its parameters.

Condition 1. Given 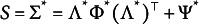 . Suppose that there exists another pair of parameters 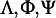 satisfy the bi-factor structure constraints, we have 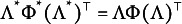 and 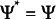 .

Condition 2.

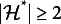
 . In addition, there exists 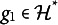 such that 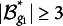 and any two rows of 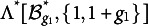 are linearly independent.

Theorem 1. Suppose that Conditions 1 and 2 hold. For any parameters 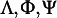 that satisfy 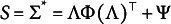 , there exist a diagonal sign-flip matrix 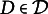 and a permutation matrix 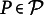 such that 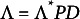 and 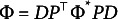 .

The proof of Theorem 1 is given in Section G.1 of the Supplementary Material.

Remark 1. We note that without additional information, the best we can achieve is to recover 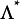 and 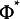 up to 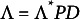 and 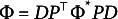 , where the permutation matrix *P* and sign-flip matrix *D* are necessary to account for the label and sign indeterminacies of the factor model. In that case, 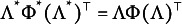 , and thus, the model implied covariance matrix is the same. A similar indeterminacy issue also appears in exploratory factor analysis (see, e.g., Remark 1 in Liu et al., 2023).

Remark 2. Condition 1 ensures the separation between low rank matrix 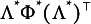 and diagonal matrix 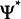 . A sufficient condition for Condition 1 that can be easily checked in practice is given in Condition 3 below, which requires that each group has at least three nonzero group loadings and there exist at least three groups whose group loadings are linearly independent of the corresponding common loadings. We refer to Theorem 5.1 in Anderson and Rubin (1956) and Theorem 2 in Fang et al. (2021) for alternative sufficient conditions of Condition 1.

Condition 3.

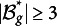
 for all 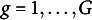 and 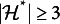 .

Remark 3. Condition 2 is similar to Condition E3S for Proposition 1 in Fang et al. (2021), where the latter is used to ensure the identifiability of parameters when the bi-factor structure is known. It is a sufficient condition that ensures if there is a pair of 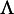 and 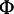 that also satisfies the constraints of a bi-factor model and 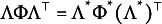 , then there must be a permutation matrix *P* and sign-flip matrix *D* such that 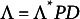 and 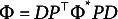 . This condition first requires the existence of at least two group factors, for each of which the group loadings are linearly independent of the corresponding common loadings. It further requires that there exists a group factor 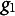 among these group factors, such that (1) 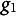 has at least three nonzero group loadings, and (2) any two-by-two submatrix of 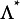 , whose rows correspond to any two variables loading on 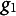 and columns correspond to the common factor and the group factor 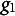 , is of full rank. We note that the requirement of Condition 2 is very mild. In fact, the set of parameters not satisfying this condition has zero Lebesgue measure in the parameter space for bi-factor models satisfying that there are at least two group factors 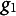 and 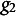 such that 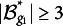 and 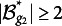 .

### 2.2 Proposed ALM

Following the previous discussion, we see that we can perform exploratory bi-factor analysis by solving the optimization problem with some equality constraints and the constraint that 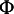 is a correlation matrix. To deal with the constraints in 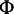 , we consider a reparameterization of 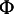 based on a Cholesky decomposition, where the explicit form of the reparameterization is given in Section A of the Supplementary Material. With slight abuse of notation, we reexpress the covariance matrix as 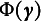 , where 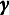 is a 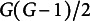 dimensional unconstrained parameter vector. In addition, we use 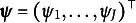 to denote the vector of diagonal entries of 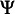 and reexpress the residual covariance matrix as 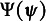 . Thus, the optimization problem (2) is now simplified as 
(3)

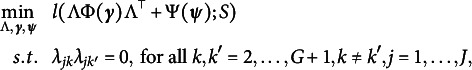

which is an equality-constrained optimization problem.

The standard approach for solving such a problem is the ALM (e.g., Bertsekas, 2014). This method aims to find a solution to (3) by solving a sequence of unconstrained optimization problems. Let *t* denote the *t*th unconstrained optimization problem in the ALM. The corresponding objective function, also known as the augmented Lagrangian function, takes the form 
(4)

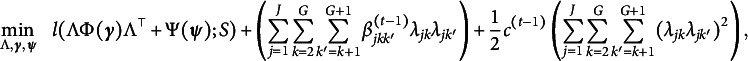

where 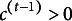 and 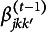 s are auxiliary coefficients of the ALM determined by the initial values when 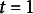 and the previous optimization when 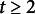 . Details of the ALM are given in Algorithm 1, in which the function *h* returns the second-largest value of a vector.

The ALM can be seen as a penalty method for solving constrained optimization problems. It replaces a constrained optimization problem with a series of unconstrained problems. It adds a penalty term, that is, the third term in (4), to the objective to enforce the constraints. The tuning parameter 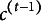 can be seen as the weight of the penalty term. In fact, as 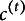 goes to infinity while 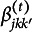 s remain bounded, the solution has to converge to one satisfying the equality constraints in (3), as otherwise, the objective function value in (4) will diverge to infinity. However, the ALM is not purely a penalty method in the sense that it also adds the second term in (4) to mimic a Lagrange multiplier (see, e.g., Chapter 12, Nocedal & Wright, 1999), for which 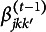 s are the weights. An advantage of the ALM is that, with the inclusion of the Lagrangian term (i.e., the second term), the method is guaranteed to converge to a local solution satisfying the equality constraints without requiring 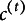 to go to infinity. This is important, as when 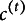 is very large, the optimization problem (4) becomes ill-conditioned and thus hard to solve.

The updating rule of 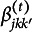 and 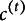 follows equations (1) and (47) in Chapter 2.2 of Bertsekas (2014). The updating rule for 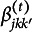 follows the first-order optimality condition based on optimizations (3) and (4). The updating rule for 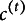 ensures that it will become sufficiently large, which is necessary to guarantee the solution of the algorithm to converge to the feasible region defined by the zero constraints. On the other hand, it also prevents 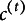 from growing too quickly with which the optimization (4) is ill-conditioned. As shown in Chapter 2.2 of Bertsekas (2014), as long as the sequences 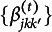 remain bounded, the sequence 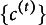 remains bounded. We follow the recommended choices of 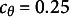 and 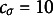 in Bertsekas (2014), while pointing out that the performance of Algorithm 1 is quite robust against the choices of these tuning parameters (see Section C of the Supplementary Material for a sensitivity analysis). The convergence of Algorithm 1 is guaranteed by Proposition 2.7 of Bertsekas (2014).

We remark on the stopping criterion in the implementation of Algorithm 1. We monitor the convergence of the algorithm based on two criteria: (1) the change in parameter values in two consecutive steps, measured by 

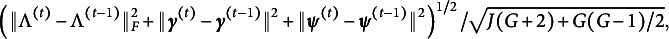



**Figure:**
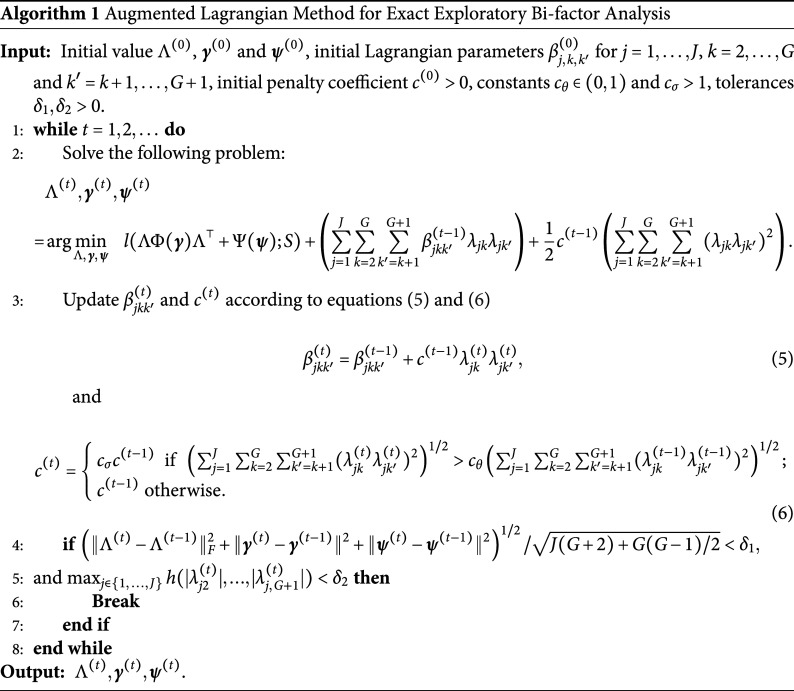

where 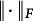 denotes the Frobenius norm of a matrix and 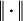 denotes the standard Euclidian norm, and (2) the distance between the estimate and the space of bi-factor loading matrices measured by 

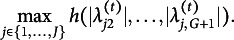

We stop the algorithm when both criteria are below their pre-specified thresholds, 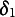 and 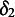 , respectively. The first criterion is a standard criterion for monitoring parameter convergence. This criterion being sufficiently small suggests the convergence of the algorithm. The second criterion is used to ensure that the solution is sufficiently close to the feasible set of optimization defined by the equality constraints. This criterion being below 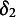 means that for each variable *j*, there can only be one group loading whose absolute value is above the threshold 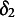 , and all the rest have absolute values below the threshold. Based on this, we can obtain an estimate of the bi-factor structure. More specifically, let *T* be the last iteration number. Then the estimated bi-factor model structure is given by 

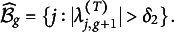

By our choice of the stopping criterion, the resulting 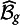 , 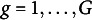 , gives a partition of all the variables, and thus, the bi-factor structure is satisfied. For simulation studies in Section 3, we choose 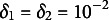 . For real data analysis in Section 4, we choose 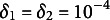 to get a more accurate and reliable result.

The optimization problem (4) is non-convex and can get stuck in a local minimum. Thus, we recommend running the proposed algorithm multiple times with random starting points and choosing the solution with the smallest objective function value. The algorithm can also suffer from slow convergence, especially when the penalty term becomes large. When the algorithm does not converge within 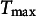 iterations, we suggest using the estimated parameters at the 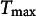 th iteration as the initial parameters and restarting the optimization until a good proportion of them converge. In the simulation study in Section 3, the estimated parameters obtained using 50 random starting points are close to the global minimum in most cases in the simulation study. For the real data example in Section 4, 200 random starting points are used to ensure a reliable result. We set 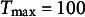 in all of our numerical studies.

### 2.3 Selecting the number of group factors

In Sections 2.1 and 2.2, the number of group factors *G* is treated as known. In practice, we can select its value based on the BIC (Schwarz, 1978). Let 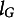 denote the minimum loss function value in (2) when the number of group factors is *G*. As 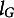 differs from twice the negative log-likelihood of the bi-factor model with *G* group factors by a constant, and the numbers of nonzero parameters in 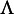 and 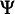 do not depend on *G*, it is not difficult to see that the BIC of the bi-factor model with *G* group factors differs from 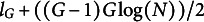 by a constant. Note that 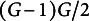 is the number of free parameters in the correlation matrix 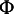 . Thus, we write 

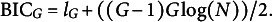



In practice, we choose the number of group factors *G* from a candidate set 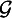 . For each value of 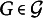 , we run the ALM described in Section 2.2 to obtain the value of 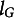 . We then compute 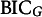 and choose 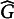 with the smallest BIC value, that is, 

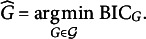



## 3 Simulation study

### 3.1 Study I

In this study, we compare the proposed method with the oblique bi-factor rotation in Jennrich and Bentler (2012) regarding their performance in the estimation accuracy of parameters and the recovery of the bi-factor structure. We consider two different settings for the data generation mechanism: (1) the observed data are generated from an exact bi-factor model, and (2) the observed data are generated from an approximate bi-factor model, where the loading matrix is generated by adding small perturbations to an exact bi-factor loading matrix.

The oblique bi-factor rotation method first estimates the loading matrix 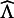 under the exploratory factor analysis setting by the optimization problem 
(7)

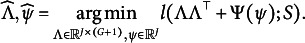

We restrict 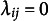 for 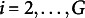 and 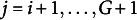 to avoid the rotational indeterminacy of 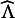 , as suggested in Anderson and Rubin (1956). Then the rotated solutions 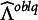 and 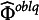 are obtained by finding a rotation matrix that solves the optimization problem for oblique bi-factor rotation (Jennrich & Bentler, 2012). The implementation in the R package GPArotation (Bernaards & Jennrich, 2005) is used for solving this optimization problem, which is based on a gradient projection algorithm. The optimization problem for rotation is also non-convex and thus may converge to local solutions. For a fair comparison, we also use 50 random starting points for the initial rotation matrix, which is the same as the number of random starting points that are used when running Algorithm 1.

We first examine the accuracy in estimating the loading matrix. We calculate the mean squared error (MSE) for 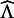 , after adjusting for the label and sign indeterminacy as considered in Theorem 1 and further discussed in Remark 1. More specifically, let 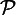 and 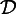 be the sets of permutation and sign flip matrices, respectively, as defined in Theorem 1. We define the MSE for 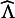 as 

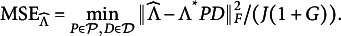

Note that when data are generated from an approximate bi-factor model, 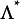 does not have an exact bi-factor structure. This MSE is calculated for the loading matrix estimates from both methods.

To compare the two methods in terms of their performance in recovering the bi-factor structure, we derive a sparse loading structure from the rotated solution by hard thresholding, a procedure also performed in Jennrich and Bentler (2012) for examining structure recovery. We let 

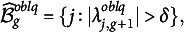

for 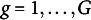 and some hard thresholding parameter 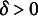 . In the analysis below, we consider three choices of hard thresholding parameter 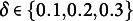 . We note that 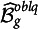 , 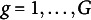 , may not yield an exact bi-factor structure as it is not guaranteed to return only one nonzero group loading parameter for each variable.

Let 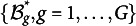 be the true nonoverlapping clusters of the *J* variables, and let 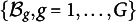 be their estimates, either from the proposed method or the rotation method. When data are generated from an approximate bi-factor model, the true group clusters 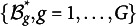 are based on the corresponding bi-factor loading matrix before the perturbation. As the group factors can only be recovered up to label swapping, as Theorem 1 suggests, we measure the matching between the true and estimated structure up to a permutation of the factor labels. Specifically, the following two evaluation criteria are considered:

- Exact Match Criterion (EMC): 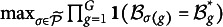 , which equals 1 when the bi-factor structure is correctly learned and 0 otherwise. Here, 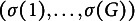 is a permutation of 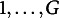 , and 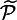 is the set of all such permutations.
- Average Correctness Criterion (ACC): 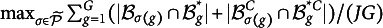 , which is the proportion of correctly identified zero and nonzero group loadings. Here, for any set 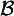 , 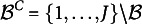 is the complement of set 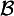 .

Here, the EMC measures the perfect recovery of the true bi-factor structure, while the ACC can be viewed as a smooth version of EMC that measures the level of partial recovery. EMC = 1 when ACC = 1 and, EMC = 0 when ACC 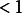 . A larger value of ACC indicates a higher level of partial recovery of the true bi-factor structure. More specifically, for a given permutation 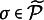 , the quantity 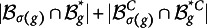 computes the number of correctly identified nonzero and zero loadings for group factor *g*. For example, consider a case with 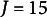 items and 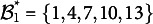 for the first group factor. If 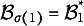 , then for the first group factor, we have 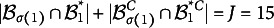 , that is, all the nonzero and zero loadings have been correctly identified. If, instead, 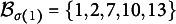 , then 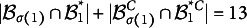 , that is, 13 out of the 15 nonzero and zero loadings have been correctly identified. The quantity 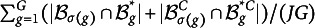 thus computes the proportion of correctly identified zero and nonzero group loadings under the given permutation 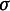 . The ACC considers all possible permutations of the group factor labels to account for the label indeterminacy.

To examine the recovery of the bi-factor structure, we consider 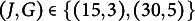 and 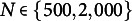 . These choices, combined with the two settings for the data generation mechanism, lead to eight simulation settings. For each setting, we let 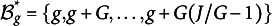 for 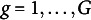 , 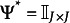 , and 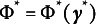 follow the reparameterization in Section 2.2, where the entries of 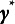 are i.i.d., following a Uniform 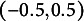 distribution. Under the settings where data are generated from an exact bi-factor model, we generate the true loading matrix 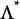 by 
(8)

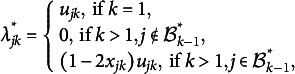

for 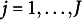 and 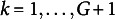 . In (8), 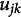 s are i.i.d., following a Uniform 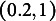 distribution, and 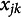 s are i.i.d., following a Bernoulli 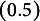 distribution. Under the settings where data are generated from an approximate bi-factor model, we generate the true loading matrix 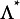 by 
(9)

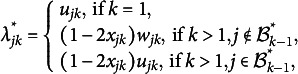

for 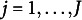 and 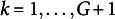 . Here, 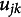 s and 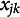 s are generated in the same way as those in the exact bi-factor model, and 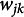 are i.i.d., following a Uniform 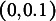 distribution. In (9), the nonzero values of 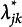 when 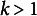 and 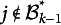 represent the perturbation of 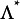 from an exact bi-factor structure.

For each setting, we first generate 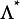 and 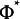 once and use them to generate 100 datasets. The true parameter values for these simulations are given in Section B of the Supplementary Material. The results about the estimation of the loading matrix are shown in Table 1. When data are generated from an exact bi-factor model, the proposed method outperforms the rotation method in terms of the MSE of the estimated loading matrix, as shown in Table 1(a). When data are generated from an approximate bi-factor model, as shown in Table 1(b), the proposed method is slightly better under the small-sample settings when 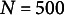 but slightly worse under the large-sample settings when 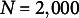 . The disadvantage of the proposed method under the large-sample settings is due to the bias brought by model misspecification. That is, the data generation model is not an exact bi-factor model, while the proposed method restricts its estimates in the space of exact bi-factor models.

**Table 1:** Simulation results of the MSE of 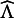 estimated by the proposed ALM method and the exploratory bi-factor rotation method

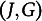	*N*	ALM	Rotation
(a) The exact bi-factor model cases.			
(15,3)	500	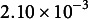	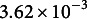
	2,000	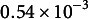	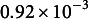
(30,5)	500	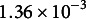	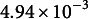
	2,000	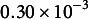	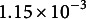
(b) The approximate bi-factor model cases.			
(15,3)	500	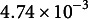	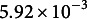
	2,000	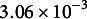	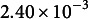
(30,5)	500	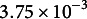	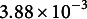
	2,000	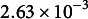	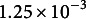

The results about the recovery of the bi-factor structure based on the EMC and ACC metrics are shown in Tables 2 and 3, respectively. For the rotation method, the threshold 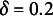 yields the best results among the three threshold choices under all the simulation settings and for both performance metrics. However, even the results of the rotation method under this choice of threshold are not as good as those from the proposed method, especially when we look at the EMC metric. For example, when 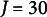 , 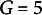 , and 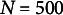 , the rotation method with 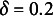 can only correctly recover the entire bi-factor structure 15 times among 100 simulations, while the proposed method can correctly recover it 85 times.

**Table 2:** Simulation results of the EMC of the proposed ALM method and the exploratory bi-factor rotation method with three choices of hard thresholding parameter 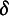

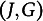	*N*	ALM	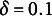	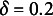	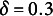
(a) The exact bi-factor model cases.					
(15,3)	500	1.00	0.18	0.90	0.28
	2,000	1.00	0.99	1.00	0.50
(30,5)	500	0.85	0.00	0.15	0.00
	2,000	1.00	0.55	0.68	0.00
(b) The approximate bi-factor model cases.					
(15,3)	500	0.99	0.00	0.62	0.33
	2,000	1.00	0.02	0.94	0.67
(30,5)	500	0.86	0.00	0.23	0.00
	2,000	1.00	0.00	0.82	0.00

**Table 3:** Simulation results of the ACC of the proposed ALM method and the exploratory bi-factor rotation method with three choices of hard thresholding parameter 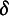

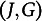	*N*	ALM	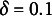	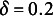	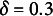
(a) The exact bi-factor model cases.					
(15,3)	500	1.000	0.966	0.998	0.976
	2,000	1.000	0.999	1.000	0.987
(30,5)	500	0.998	0.892	0.987	0.973
	2,000	1.000	0.996	0.998	0.980
(b) The approximate bi-factor model cases.					
(15,3)	500	0.999	0.864	0.989	0.978
	2,000	1.000	0.928	0.999	0.991
(30,5)	500	0.998	0.848	0.989	0.978
	2,000	1.000	0.927	0.999	0.980

**Table 4:** Simulation results of the selection of the number of factors by BIC

		ALM		Exploratory	
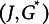	*N*	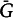	SC	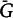	SC
(15,3)	500	3	1	3	1
	2,000	3	1	3	1
(30,5)	500	5.02	0.98	4	0
	2,000	5	1	4.99	0.99

### 3.2 Study II

In this study, we examine the selection of the number of factors by 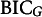 in Section 2.3. We compare it with selecting the number of factors under the exploratory factor analysis model without assuming a bi-factor structure. For the proposed method, we set the candidate set 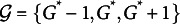 , where 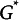 is the true number of group factors. For exploratory factor analysis, we also use the BIC for determining the number of factors, which is defined as 

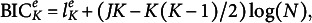

where *K* is the number of factors in the exploratory factor analysis model, and 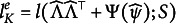 with 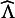 and 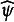 from (7). As the number of factors in the exploratory factor analysis model equals the number of group factors plus one, we choose *K* from the candidate set 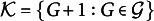 . Let 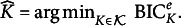 Then the estimate of *G* by exploratory factor analysis is 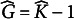 . The selection accuracy is evaluated by the selection correctness (SC) criterion, defined as 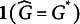 , where 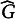 is obtained using the proposed method in Section 2.3 or under the exploratory factor analysis model described above.

We conduct simulations under four settings, with 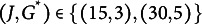 and 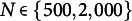 and the data generation models being the same exact bi-factor models in Study I. For each setting, 100 independent simulations are performed. The results are given in Table 4, where the column indicated by 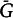 reports the average value of 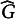 . We see that both methods can select the number of factors reasonably well, with their accuracy being 100% when 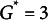 for both sample sizes. When 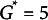 and the sample size 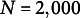 , the proposed method achieves an accuracy of 100%, and the exploratory factor analysis method achieves an accuracy of 99%. This is not surprising, given that the BIC has asymptotic consistency in selecting the number of factors under both models. It is worth noting that, when 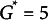 and for the smaller sample size 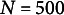 , which is the most challenging setting, the proposed method achieves an accuracy of 98%, while that of the exploratory factor analysis method is zero. More precisely, the exploratory factor analysis method selects 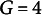 in all the replications. It suggests that the proposed method has an advantage in smaller sample settings. This result is expected, as the exploratory factor analysis method doesn’t utilize the information about the bi-factor structure. Consequently, it overestimates the number of parameters, which leads to a larger penalty term and, subsequently, a tendency to under-select *G*.

## 4 Real data analysis

In this section, we apply the exact exploratory bi-factor analysis to a personality assessment dataset based on the International Personality Item Pool (IPIP) NEO 120 personality inventory (Johnson, 2014).^1^ We investigate the structure of the Extraversion scale based on a sample of 1,107 U.K. male participants aged between 25 and 30 years. This scale consists of 24 items, which are designed to measure six facets of Extraversion, including Friendliness (E1), Gregariousness (E2), Assertiveness (E3), Activity Level (E4), Excitement-Seeking (E5), and Cheerfulness (E6) (see Section E of the Supplementary Material for the details). All the items are on a 1–5 Likert scale, and the reversely worded items have been reversely scored so that a larger score always means a higher level of extraversion. There is no missing data. Detailed descriptions of the items can be found in the Section E of the Supplementary Material.

Using a candidate set 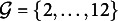 , the BIC procedure given in Section 2.3 selects seven group factors. The estimated loading matrix is given in Table 5, and the estimated factor correlation matrix is given below. The estimated model fits the data well, as implied by the commonly used fit statistics, including RMSEA = 0.044, SRMR = 0.031, CFI = 0.965, and TLI = 0.953. We point out that the estimated bi-factor structure does not meet Condition 3, one of the sufficient conditions for Theorem 1. However, as shown in Section G.2 of the Supplementary Material, with some additional mild assumptions, this structure and its parameters are still identifiable.

**Table 5:** Estimated bi-factor loading matrix 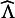 with seven group factors

Items	Sign	General	G1	G2	G3	G4	G5	G6	G7
1	+E1	0.85	0.26	0	0	0	0	0	0
2	+E1	0.73	0.48	0	0	0	0	0	0
3	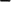 E1	0.74	0.57	0	0	0	0	0	0
4	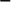 E1	0.68	0.58	0	0	0	0	0	0
5	+E2	0.94	0	0	0	0	0	0.26	0
6	+E2	1.01	0	0	0	0.17	0	0	0
7	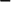 E2	0.53	0.52	0	0	0	0	0	0
8	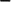 E2	0.67	0	0	0	0	0	1.06	0
9	+E3	0.37	0	0	0	0.86	0	0	0
10	+E3	0.38	0	0	0	0.81	0	0	0
11	+E3	0.28	0	0	0	0.74	0	0	0
12	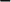 E3	0.39	0	0	0	0.75	0	0	0
13	+E4	0.20	0	0	0	0	0.81	0	0
14	+E4	0.40	0	0	0	0	0.82	0	0
15	+E4	0.40	0	0	0	0	0.60	0	0
16	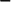 E4	0.04	0	0	0	0	0.47	0	0
17	+E5	0.46	0	0	0	0	0	0	0.46
18	+E5	0.47	0	0	0	0	0	0	0.71
19	+E5	0.35	0	0.86	0	0	0	0	0
20	+E5	0.56	0	0.71	0	0	0	0	0
21	+E6	0.59	0	0	0.42	0	0	0	0
22	+E6	0.64	0	0	0.48	0	0	0	0
23	+E6	0.46	0	0	0.76	0	0	0	0
24	+E6	0.41	0	0	0.74	0	0	0	0

We now examine the estimated model. We first notice that the loadings on the general factor are all positive. Consequently, this factor can be easily interpreted as the general extraversion factor. The seven group factors are closely related to the six aspects of extraversion. Specifically, we interpret the group factors G1, G3, G4, and G5 as the Friendliness, Cheerfulness, Assertiveness, and Activity Level factors, respectively, as the items loading on them highly overlap with the items that are used to define the corresponding aspects. In particular, the items loading on G3 and G5 are exactly those that define the Cheerfulness and Activity Level aspects, respectively. The items loading on G1 include all the items that define the Friendliness aspect and an additional item “7. Prefer to be alone,” a negatively worded item that is used to define the Gregariousness aspect. This additional item aligns well with the Friendliness dimension, given the social nature behind it. In addition, the items loading on G4 consist of all the items that define the Assertiveness aspect and an additional item “6. Talk to a lot of different people at parties,” which is used to define the Gregariousness aspect. This additional item aligns with the Assertiveness dimension in that talking to many different people at parties typically requires sufficient confidence, a key element of Assertiveness. 

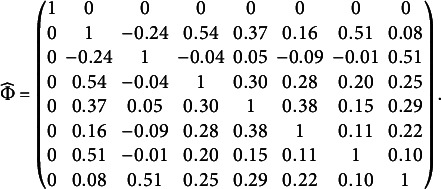



The group factors G2 and G7 may be viewed as two subdimensions of the Excitement-Seeking aspect, as each of them is loaded with two items that define the Excitement-Seeking aspect. Specifically, G2 is loaded with the items “19. Enjoy being reckless” and “20. Act wild and crazy,” while G7 is loaded with the items “17. Love excitement” and “18. Seek adventure.” We believe that G2 emphasizes the thrill of the moment of excitement and the disregard for potential consequences, while G7 emphasizes the pursuit of meaningful and fulfilling experiences. Therefore, we interpret G2 as the Reckless Excitement-Seeking factor, while interpret G7 as the Meaningful Excitement-Seeking factor. Finally, G6 is loaded with two items, “5. Love large parties” and “8. Avoid crowds,” where item 8 is reversely worded. Both items are used to define the Gregariousness aspect. Compared with items 6 and 7, which are also used to define the Gregariousness aspect but now load on two different group factors, these two items may better reflect the essence of Gregariousness—the tendency to enjoy the company of others. We thus interpret G6 as the Gregariousness factor. We also notice that most correlations between the group factors are positive, except for some of the correlations with G2. Specifically, we see that G2 (Reckless Excitement-Seeking) has a moderate negative correlation with G1 (Friendliness) while a reasonably high correlation with G7 (Meaningful Excitement-Seeking).

We have also applied the bi-factor rotation method of Jennrich and Bentler (2012) to the same data, which gives a solution with seven group factors. The resulting bi-factor structure is similar to that given by the proposed method, except that the rotation solution does not seem to contain a clear Friendliness factor (see Section F of the Supplementary Material for further details).

## 5 Discussions

This article proposes a constraint-based optimization method for exploratory bi-factor analysis. This method turns the problem of exploratory bi-factor analysis into an equality-constrained optimization problem in a continuous domain and solves this optimization problem by an ALM. Compared with the rotation method of Jennrich and Bentler (2011, 2012), the proposed method can learn an exact loading structure without a post-hoc treatment step. In the simulation studies, the ALM method achieves higher estimation accuracy when data are generated from an exact bi-factor model. In addition, it has a higher chance of recovering the true bi-factor structure than the rotation method, whether data are generated from an exact or approximate bi-factor model. Moreover, the ALM method correctly estimates the number of the group factors in most of the simulation replications. In the real data analysis concerning an Extraversion personality scale, the ALM method yields a bi-factor structure with seven group factors. The identified group factors are psychologically interpretable.

An innovation of current research is turning a model selection problem, which is combinatory by nature, into a continuous optimization problem. This avoids a computationally intensive search procedure for fitting many possible models and comparing their fits, noting that the number of possible models grows exponentially with *J*. We admit that this continuous optimization formulation also has a limitation. The space for the bi-factor loading matrix characterized by the nonlinear equality constraints in (1) is highly non-convex, and thus, the ALM may sometimes converge to a local minimum. To alleviate this issue, we suggest running the ALM with multiple random starting points and then choosing the solution with the smallest objective function value. Based on our simulation results, using 50 starting points seems sufficient to converge to somewhere close to the true parameters up to a label swapping of the group factors and a sign indeterminacy of loadings in almost all replications under the settings considered in the simulation study.

This research leads to several new directions for exploratory analysis of factor models with structure constraints on the loading matrix. First, as pointed out earlier, the proposed approach can be easily adapted to nonlinear bi-factor models for dichotomous, ordinal, and nominal data. Under the confirmatory setting, these models are typically estimated by maximizing the marginal log-likelihood function or other objective functions (e.g., a composite likelihood). Under the exploratory setting, one only needs to maximize the same objective function subject to the same bi-factor constraints in (1), for which the ALM adapts naturally. It is worth noting that, however, the marginal likelihood of the nonlinear bi-factor models typically involves multidimensional integrals with respect to the factors, and they do not have an analytic form. Consequently, solving the Lagrangian augmented objective functions using the standard expectation-maximization (EM) algorithm (Bock & Aitkin, 1981; Dempster et al., 1977) can be computationally intensive. One possible solution is to use a stochastic approximation method (Zhang & Chen, 2022). These methods avoid the high computational cost of numerical integrals in the EM algorithm by constructing stochastic gradients of the marginal log-likelihood through Markov chain Monte Carlo sampling.

Second, the proposed constraints can also be combined with exploratory factor analysis techniques to learn a bi-factor structure in two steps. Suppose an initial loading matrix estimate 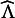 has been obtained under the constraint that the factors are orthogonal (i.e., 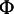 is an identity matrix). It may be obtained by a standard exploratory factor analysis method. In that case, we can find a bi-factor structure that best approximates 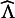 (up to a rotation) by minimizing 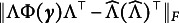 with respect to 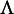 and 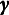 under the constraints in (1). This optimization can again be solved by an ALM.

Third, as we demonstrate in Section D of the Supplementary Material, the set of constraints in (1) can be extended to characterize the loading structure of a hierarchical factor model (Schmid & Leiman, 1957; Yung et al., 1999), which can be used to learn a hierarchical factor structure. This exploratory hierarchical factor analysis may allow researchers to learn more refined and interpretable latent structures from psychometric data. However, one should note that exploratory hierarchical factor analysis is more complex than exploratory bi-factor analysis, as the factor hierarchy in the former can be much more complex than the two-layer hierarchy in the latter. The learning algorithm in Section D of the Supplementary Material requires the factor hierarchy to be known (see, e.g., Panel (b) of Figure D.1 in the Supplementary Material). The problem becomes more challenging when the factor hierarchy is unknown, in which case we need to learn both the factor hierarchy and the loading pattern of the variables. We leave this problem for future investigation.

Finally, we point out that the proposed method always returns an estimated bi-factor model, whether it fits the data or not. The simulation study in Section 3 shows that the proposed method has robust performance when data are generated by an approximate bi-factor model. However, under more general settings, it remains to test the goodness-of-fit of the estimated model to decide whether a bi-factor model suits the data. If the bi-factor model does not fit the data well, we may consider a more flexible factor model. For example, we may apply the bi-factor rotation method or a rotation method for traditional exploratory factor analysis to allow for more cross-loadings. Alternatively, we may learn approximate bi-factor models in an exploratory manner by relaxing the equality constraints in (2) with inequality constraints in the form of 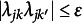 , for all 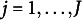 , and 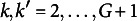 , 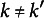 , where 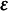 is a tuning parameter that controls the level of approximation to a bi-factor model. A larger value of 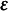 leads to a more flexible model space and, thus, a more satisfactory fit, while a smaller value of 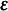 leads to a better approximation to a bi-factor model that may have better interpretability. In this sense, 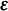 provides a trade-off between model goodness-of-fit and bi-factor interoperability. This inequality-constrained optimization may be solved using an interior-point method, which can incorporate the inequality constraints through suitable barrier functions (e.g., log-barriers). We leave this idea for future investigation.

## Supporting information

Qiao et al. supplementary material Qiao et al. supplementary material

## Data Availability

Code implementing the proposed method is open-source and publicly available at https://github.com/EmetSelch97/Eebf.

## Footnotes

1 The data are downloaded from https://osf.io/tbmh5/.

## Supplementary material

The supplementary material for this article can be found at https://doi.org/10.1017/psy.2025.17.
