# Supplementary material for: Exact Exploratory Bi-factor Analysis: A Constraint-Based Optimization Approach
Source: Psychometrika. 2025 May 16;90(3):998–1013. doi: 10.1017/psy.2025.17 (PMC12483720; doi:10.1017/psy.2025.17)
Supplement: Qiao et al. supplementary material [file S0033312325000171sup001.pdf]

Appendix

A Reparameterization of Φ

To deal with the constraints in Φ, we consider the following reparameterisation that has been considered in Alfonzetti et al. (2024), which is also similar to the implementation in the state-of-the-art statistical software Stan (Stan Development Team, 2022):

$$\Phi = \left(\begin{bmatrix} 1 & \mathbf{0}^\top \\ \mathbf{0} & U^T \end{bmatrix} \right) \left(\begin{bmatrix} 1 & \mathbf{0}^\top \\ \mathbf{0} & U \end{bmatrix} \right),$$

where U is defined recursively by

$$U_{ij} = \begin{cases} 0 \text{ if } i > j; \\ 1 \text{ if } i = j = 1; \\ z_{ij} \text{ if } 1 = i < j; \\ \frac{z_{ij}}{z_{(i-1)j}} U_{(i-1)j} (1 - z^2_{(i-1)j})^{1/2} \text{ if } 1 < i < j; \\ \frac{U_{(i-1)j}}{z_{(i-1)j}} (1 - z^2_{(i-1)j})^{1/2} \text{ if } 1 < i = j. \end{cases}$$

Here $z_{ij} = \tanh(\gamma_{ij})$ is the Fisher's transformation of $G(G-1)/2$ unconstrained parameters γ_{ij}.

B Population Parameter Values in Simulations

In this section, we supplement the population values of factor loadings and factor correlations in Section 3. Under the setting $(J, G) = (15, 3)$, the loading matrix Λ^* and Φ^* in (8) and (9) are given in (B.1), (B.2),

(B.3) and (B.4) respectively.

$$\Lambda^* = \begin{pmatrix} 0.36 & -0.60 & 0 & 0 \\ 0.89 & 0 & 0.64 & 0 \\ 0.94 & 0 & 0 & 0.92 \\ 0.90 & 0.33 & 0 & 0 \\ 0.45 & 0 & -0.90 & 0 \\ 0.24 & 0 & 0 & 0.86 \\ 0.45 & -0.60 & 0 & 0 \\ 0.74 & 0 & -0.75 & 0 \\ 0.64 & 0 & 0 & -0.60 \\ 0.50 & -0.83 & 0 & 0 \\ 0.54 & 0 & -0.43 & 0 \\ 0.43 & 0 & 0 & 0.86 \\ 0.46 & -0.33 & 0 & 0 \\ 0.67 & 0 & 0.34 & 0 \\ 0.90 & 0 & 0 & -0.83 \end{pmatrix} \tag{B.1}$$

$$\Phi^* = \begin{pmatrix} 1 & 0 & 0 & 0 \\ 0 & 1 & 0.29 & 0.11 \\ 0 & 0.29 & 1 & 0.01 \\ 0 & 0.11 & 0.01 & 1 \end{pmatrix} \tag{B.2}$$

$$\Lambda^* = \begin{pmatrix} 0.36 & -0.6 & -0.05 & 0.06 \\ 0.89 & 0.04 & 0.64 & 0.09 \\ 0.94 & -0.09 & -0.08 & 0.92 \\ 0.90 & 0.33 & 0.02 & -0.09 \\ 0.45 & -0.07 & -0.90 & -0.06 \\ 0.24 & -0.02 & 0.09 & 0.86 \\ 0.45 & -0.60 & -0.09 & 0.02 \\ 0.74 & 0.05 & -0.75 & 0.09 \\ 0.64 & 0.05 & -0.02 & -0.60 \\ 0.50 & -0.83 & 0.05 & 0.06 \\ 0.54 & 0.02 & -0.43 & 0.07 \\ 0.43 & 0.03 & -0.09 & 0.86 \\ 0.46 & -0.33 & 0.00 & 0.04 \\ 0.67 & 0.05 & 0.34 & 0.04 \\ 0.90 & 0.00 & -0.06 & -0.83 \end{pmatrix} \tag{B.3}$$

$$\Phi^* = \begin{pmatrix} 1 & 0 & 0 & 0 \\ 0 & 1 & -0.22 & -0.07 \\ 0 & -0.22 & 1 & 0.46 \\ 0 & -0.07 & 0.46 & 1 \end{pmatrix} \tag{B.4}$$

Under the setting $(J, G) = (30, 5)$, the loading matrix Λ in (8) and (9) are given in (B.5) and (B.6). The

correlation matrix Φ in (8) and (9) are given in (B.7) and (B.8).

$$\Lambda^* = \begin{pmatrix}
0.40 & -0.90 & 0 & 0 & 0 & 0 \\
0.53 & 0 & -0.62 & 0 & 0 & 0 \\
0.21 & 0 & 0 & -0.78 & 0 & 0 \\
0.69 & 0 & 0 & 0 & -0.81 & 0 \\
0.57 & 0 & 0 & 0 & 0 & 0.90 \\
0.91 & -0.93 & 0 & 0 & 0 & 0 \\
0.31 & 0 & -0.47 & 0 & 0 & 0 \\
0.41 & 0 & 0 & -0.79 & 0 & 0 \\
0.31 & 0 & 0 & 0 & -0.35 & 0 \\
0.90 & 0 & 0 & 0 & 0 & -0.59 \\
0.59 & 0.80 & 0 & 0 & 0 & 0 \\
0.83 & 0 & -0.96 & 0 & 0 & 0 \\
0.84 & 0 & 0 & -0.89 & 0 & 0 \\
0.29 & 0 & 0 & 0 & -0.23 & 0 \\
0.90 & 0 & 0 & 0 & 0 & 0.77 \\
0.68 & -0.56 & 0 & 0 & 0 & 0 \\
0.95 & 0 & 0.89 & 0 & 0 & 0 \\
0.87 & 0 & 0 & 0.61 & 0 & 0 \\
0.45 & 0 & 0 & 0 & 0.41 & 0 \\
0.52 & 0 & 0 & 0 & 0 & 0.51 \\
0.43 & -0.42 & 0 & 0 & 0 & 0 \\
0.43 & 0 & -0.30 & 0 & 0 & 0 \\
0.34 & 0 & 0 & -0.31 & 0 & 0 \\
0.73 & 0 & 0 & 0 & 0.53 & 0 \\
0.69 & 0 & 0 & 0 & 0 & -0.83 \\
0.30 & 0.34 & 0 & 0 & 0 & 0 \\
0.25 & 0 & -0.60 & 0 & 0 & 0 \\
0.58 & 0 & 0 & 0.37 & 0 & 0 \\
0.28 & 0 & 0 & 0 & -0.28 & 0 \\
0.85 & 0 & 0 & 0 & 0 & 0.82
\end{pmatrix} \tag{B.5}$$

$$
\Lambda^* = \begin{pmatrix}
0.40 & -0.90 & -0.07 & 0.01 & 0.01 & 0.02 \\
0.53 & 0.02 & -0.62 & -0.06 & 0.09 & 0.00 \\
0.21 & 0.01 & -0.01 & -0.78 & -0.02 & -0.03 \\
0.69 & -0.05 & 0.01 & 0.08 & -0.81 & -0.07 \\
0.57 & 0.05 & 0.06 & -0.09 & 0.03 & 0.90 \\
0.91 & -0.93 & -0.09 & -0.01 & 0.05 & 0.04 \\
0.31 & 0.02 & -0.47 & 0.04 & 0.05 & -0.05 \\
0.41 & 0.04 & -0.07 & -0.79 & 0.02 & 0.05 \\
0.31 & 0.05 & 0.01 & -0.07 & -0.35 & -0.04 \\
0.90 & 0.05 & -0.07 & -0.10 & 0.03 & -0.59 \\
0.59 & 0.80 & -0.05 & -0.10 & -0.05 & 0.02 \\
0.83 & 0.10 & -0.96 & -0.07 & 0.06 & -0.03 \\
0.84 & 0.03 & 0.08 & -0.89 & -0.01 & -0.04 \\
0.29 & 0.09 & -0.05 & -0.01 & -0.23 & 0.04 \\
0.90 & -0.10 & -0.04 & 0.07 & -0.08 & 0.77 \\
0.68 & -0.56 & 0.02 & -0.01 & -0.07 & -0.08 \\
0.95 & 0.05 & 0.89 & -0.03 & 0.04 & -0.08 \\
0.87 & 0.09 & 0.01 & 0.61 & -0.07 & 0.05 \\
0.45 & 0.04 & -0.04 & 0.00 & 0.41 & 0.06 \\
0.52 & -0.04 & -0.05 & -0.10 & -0.03 & 0.51 \\
0.43 & -0.42 & -0.07 & -0.04 & -0.07 & -0.08 \\
0.43 & 0.02 & -0.30 & 0.05 & -0.09 & 0.06 \\
0.34 & 0.05 & -0.02 & -0.31 & 0.05 & -0.04 \\
0.73 & -0.07 & 0.10 & 0.09 & 0.53 & 0.04 \\
0.69 & -0.09 & -0.03 & -0.08 & -0.05 & -0.83 \\
0.30 & 0.34 & 0.09 & -0.04 & 0.01 & 0.01 \\
0.25 & 0.02 & -0.60 & 0.09 & 0.04 & 0.06 \\
0.58 & -0.08 & -0.03 & 0.37 & -0.02 & 0.08 \\
0.28 & -0.08 & 0.04 & -0.03 & -0.28 & -0.01 \\
0.85 & -0.06 & 0.07 & 0.08 & -0.09 & 0.82
\end{pmatrix} \tag{B.6}
$$

$$\Phi^* = \begin{pmatrix} 1 & 0 & 0 & 0 & 0 & 0 \\ 0 & 1 & -0.37 & -0.26 & -0.41 & -0.07 \\ 0 & -0.37 & 1 & -0.08 & -0.14 & -0.26 \\ 0 & -0.26 & -0.08 & 1 & 0.42 & 0.06 \\ 0 & -0.41 & -0.14 & 0.42 & 1 & 0.20 \\ 0 & -0.07 & -0.26 & 0.06 & 0.20 & 1 \end{pmatrix} \tag{B.7}$$

$$\Phi^* = \begin{pmatrix} 1 & 0 & 0 & 0 & 0 & 0 \\ 0 & 1 & -0.15 & 0.15 & 0.10 & -0.19 \\ 0 & -0.15 & 1 & 0.22 & -0.01 & -0.03 \\ 0 & 0.15 & 0.22 & 1 & -0.03 & -0.15 \\ 0 & 0.10 & -0.01 & -0.03 & 1 & 0.28 \\ 0 & -0.19 & -0.03 & -0.15 & 0.28 & 1 \end{pmatrix} \tag{B.8}$$

C Sensitivity Analysis

In this section, we carry out a sensitivity analysis on the parameters c_θ and c_σ of the proposed ALM method. We consider the same exact bi-factor model settings as in Study I of Section 3.1. For each settings, we choose $c_\theta \in \{0.25, 0.5, 0.75\}$ and $c_\sigma \in \{5, 10, 15\}$, resulting in 9 possible combinations of (c_θ, c_σ). The estimation of loading matrix, the computation time of ALM, and the results of the recovery of the bi-factor structure are shown in Table C.1 to Table C.4. From the sensitivity analysis, we can see that the ALM's results are relatively stable with respect to the choice of parameters c_θ and c_σ.

D Extension to Hierarchical Factor Analysis

D.1 Constrained Optimisation for Exploratory Hierarchical Factor Analysis

To further demonstrate the advantages of the constraint-based approach, we discuss how it can be extended for exploratory hierarchical factor analysis. Following the terminology adopted in Yung et al. (1999), we

Table C.1: Sensitivity Analysis of MSE of $\widehat{\Lambda}$.

c_σ \ c_θ	0.25	0.5	0.75
5	2.10×10^{-3}	2.10×10^{-3}	2.10×10^{-3}
10	2.10×10^{-3}	2.10×10^{-3}	2.10×10^{-3}
15	2.10×10^{-3}	2.10×10^{-3}	2.10×10^{-3}

(a) $J = 15$, $G = 3$, $n = 500$

c_σ \ c_θ	0.25	0.5	0.75
5	0.54×10^{-3}	0.54×10^{-3}	0.54×10^{-3}
10	0.54×10^{-3}	0.54×10^{-3}	0.54×10^{-3}
15	0.54×10^{-3}	0.54×10^{-3}	0.54×10^{-3}

(b) $J = 15$, $G = 3$, $n = 2000$

c_σ \ c_θ	0.25	0.5	0.75
5	1.36×10^{-3}	1.38×10^{-3}	1.39×10^{-3}
10	1.36×10^{-3}	1.42×10^{-3}	1.33×10^{-3}
15	1.42×10^{-3}	1.36×10^{-3}	1.44×10^{-3}

(c) $J = 30$, $G = 5$, $n = 500$

c_σ \ c_θ	0.25	0.5	0.75
5	0.30×10^{-3}	0.30×10^{-3}	0.30×10^{-3}
10	0.30×10^{-3}	0.30×10^{-3}	0.30×10^{-3}
15	0.30×10^{-3}	0.30×10^{-3}	0.30×10^{-3}

(d) $J = 30$, $G = 5$, $n = 2000$

Table C.2: Sensitivity Analysis of EMC.

c_σ \ c_θ	0.25	0.5	0.75
5	1.00	1.00	1.00
10	1.00	1.00	1.00
15	1.00	1.00	1.00

(a) $J = 15$, $G = 3$, $n = 500$

c_σ \ c_θ	0.25	0.5	0.75
5	1.00	1.00	1.00
10	1.00	1.00	1.00
15	1.00	1.00	1.00

(b) $J = 15$, $G = 3$, $n = 2000$

c_σ \ c_θ	0.25	0.5	0.75
5	0.86	0.86	0.85
10	0.85	0.84	0.85
15	0.83	0.86	0.83

(c) $J = 30$, $G = 5$, $n = 500$

c_σ \ c_θ	0.25	0.5	0.75
5	1.00	1.00	1.00
10	1.00	1.00	1.00
15	1.00	1.00	1.00

(d) $J = 30$, $G = 5$, $n = 2000$

Table C.3: Sensitivity Analysis of ACC.

c_σ \ c_θ	0.25	0.5	0.75
5	1.000	1.000	1.000
10	1.000	1.000	1.000
15	1.000	1.000	1.000

(a) $J = 15$, $G = 3$, $n = 500$

c_σ \ c_θ	0.25	0.5	0.75
5	1.000	1.000	1.000
10	1.000	1.000	1.000
15	1.000	1.000	1.000

(b) $J = 15$, $G = 3$, $n = 2000$

c_σ \ c_θ	0.25	0.5	0.75
5	0.998	0.998	0.998
10	0.998	0.997	0.998
15	0.997	0.998	0.997

(c) $J = 30$, $G = 5$, $n = 500$

c_σ \ c_θ	0.25	0.5	0.75
5	1.000	1.000	1.000
10	1.000	1.000	1.000
15	1.000	1.000	1.000

(d) $J = 30$, $G = 5$, $n = 2000$

Table C.4: Sensitivity Analysis of Computation time(s).

c_σ \ c_θ	0.25	0.5	0.75
5	0.13	0.13	0.13
10	0.13	0.13	0.12
15	0.10	0.09	0.08

(a) $J = 15$, $G = 3$, $n = 500$

c_σ \ c_θ	0.25	0.5	0.75
5	0.10	0.09	0.10
10	0.10	0.09	0.09
15	0.07	0.06	0.06

(b) $J = 15$, $G = 3$, $n = 2000$

c_σ \ c_θ	0.25	0.5	0.75
5	0.52	0.51	0.50
10	0.49	0.47	0.41
15	0.36	0.33	0.30

(c) $J = 30$, $G = 5$, $n = 500$

c_σ \ c_θ	0.25	0.5	0.75
5	0.38	0.37	0.37
10	0.37	0.33	0.28
15	0.23	0.22	0.24

(d) $J = 30$, $G = 5$, $n = 2000$

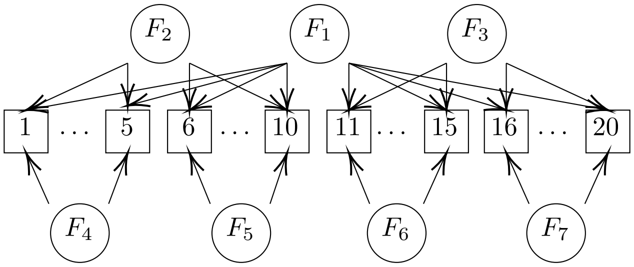

(a) The path diagram of a three-layer hierarchical factor model.

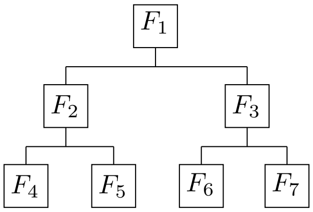

(b) The corresponding factor hierarchy.

Figure D.1: The illustrative example of a three-layer hierarchical factor model.

consider general hierarchical factor models. Such a model has several layers of factors. In each layer, each observed variable loads on exactly one of the factors in that layer. The numbering of the layers is determined by the number of factors in the layer, starting from the layer with the largest number of factors. Each factor in a lower layer is nested within a factor in a higher layer, in the sense that the variables loading on the lower-layer factor must also all load on a higher-layer factor. All the factors are assumed to be uncorrelated (i.e., Φ is an identity matrix), though this assumption may be relaxed to allow some correlations between factors within the same layer as in the extended bi-factor model.

Panel (a) of Figure D.1 provides the path diagram of a hierarchical factor model that has three layers, with factor F_1 in layer 3, factors F_2 and F_3 in layer 2, and factors F_4-F_7 in layer 1. The corresponding factor hierarchy is summarised in Panel (b) of Figure D.1 that takes the form of a tree, where F_2 and F_3 are nested within F_1, F_4 and F_5 are nested within F_2, and F_6 and F_7 are nested within F_3. In what follows, we show how the loading structure of this three-layer hierarchical model can be learned by a constrained optimisation method, assuming that the factor hierarchy in Panel (b) of Figure D.1 is known while the variables loading on each factor are unknown. The goal is to learn how the observed variables load on the seven factors.

Following the same notation for bi-factor analysis, the population covariance matrix of observed variables under the hierarchical factor model can be written as

$$\Sigma = \Lambda\Lambda^\top + \Psi,$$

where Λ is a $J \times 7$ matrix, and Ψ is a $J \times J$ diagonal matrix. Note that we no longer need the correlation

matrix Φ in the expression as it is now an identity matrix. The constraints implied by the hierarchical factor structure become:

$$\begin{aligned}&\lambda_{j2}\lambda_{j3} = 0, \quad \lambda_{j2}\lambda_{j6} = 0, \quad \lambda_{j2}\lambda_{j7} = 0,\\&\lambda_{j3}\lambda_{j4} = 0, \quad \lambda_{j3}\lambda_{j5} = 0,\\&\lambda_{j4}\lambda_{j5} = 0, \quad \lambda_{j6}\lambda_{j7} = 0, \quad j = 1, ..., J.\end{aligned} \tag{D.1}$$

Consequently, the corresponding hierarchical factor model can be learned by minimising the loss function $l(\Lambda\Lambda^\top + \Psi(\boldsymbol{\psi}); S)$, subject to the constraints in (D.1).

Although the above discussion focuses on the specific hierarchical factor structure in Figure D.1, when given a different factor hierarchy, it is easy to derive similar constraints as in (D.1) by induction. Based on the constraints, the corresponding hierarchical factor model can be learned by an ALM.

Finally, we note that the factor hierarchy is typically unknown in practice. In that case, we need an algorithm that simultaneously learns the factor hierarchy and the variable loadings on the hierarchical factors. As there are exponentially many choices for the structure of factor hierarchy, this problem is more challenging than the setting when the factor hierarchy is known. It is also more challenging than exploratory bi-factor analysis with unknown group factors, as the bi-factor model has a simple two-layer factor hierarchy that is completely determined by the number of factors.

D.2 Simulation

In this section, we examine the recovery of the hierarchical structure of our method. For $J \in \{20, 40\}$ and $N \in \{500, 2000\}$, a data generation model is considered, resulting in a total of 4 simulation settings. With slight abuse of notation, we denote by $\mathcal{B}_g^*$ as the true item groups related to the gth factor. In the data generation model, $B_1^* = \{1, \ldots, J\}$, $B_2^* = \{1, \ldots, J/2\}$, $B_3^* = \{J/2, \ldots, J\}$, $B_4^* = \{1, \ldots, J/4\}$, $B_5^* = \{J/4, \ldots, J/2\}$, $B_6^* = \{J/2, \ldots, 3J/4\}$, $B_7^* = \{3J/4, \ldots, J\}$. $\Psi^* = \mathbb{I}_{J\times J}$, and Λ^* follows

$$\lambda_{jk}^* = \begin{cases} u_{jk} \ \text{ if } \ k = 1; \\ 0 \ \text{ if } \ k > 1, j \notin \mathcal{B}_{k-1}^*; \\ (1 - 2x_{jk})u_{jk} \ \text{ if } \ k > 1, j \in \mathcal{B}_{k-1}^*, \end{cases}$$

for $j = 1, \ldots, J$ and $k = 1, \ldots, G+1$. Here, u_{jk}s are i.i.d., following a Uniform$(0.2, 1)$ distribution, and x_{jk}s are i.i.d., following a Bernoulli(0.5) distribution.

The estimated parameters $\widehat{\Lambda}$ and $\widehat{\Psi}$ follow the same ALM algorithm in Section 2.2 except that the distance

between the estimate and the space of the hierarchical factor loading matrices measured by

$$\max_{j\in\{1,...,J\}} \widetilde{h}(|\lambda_{j2}^{(t)}|, ..., |\lambda_{j,G+1}^{(t)}|),$$

where the function $\widetilde{h}$ returns the third-largest value of a vector. The estimated hierarchical factor model structure is then given by

$$\widehat{\mathcal{B}}_g = \{j : |\lambda_{j,g+1}^{(T)}| > \delta_2\}.$$

We also choose $\delta_2 = 10^{-2}$ on the following simulation study.

Since label-switching problem exists in factors that are nested within the same hierarchical factor, there exists 8 possible permutations of labels resulting in the same hierarchical structure. We denote by $\mathcal{R}$ as the set of the 8 permutations. Then, the evaluation criteria for the recovery of the hierarchical structure are defined as:

- Exact Match Criterion(EMC): $\max_{\sigma\in\mathcal{R}} \prod_{g=1}^{G} \mathbf{1}(\mathcal{B}_{\sigma(g)} = \mathcal{B}_g^*)$, which equals 1 when the bi-factor structure is correctly learned and 0 otherwise.
- Average Correctness Criterion(ACC): $\max_{\sigma\in\mathcal{R}} \sum_{g=1}^{G} (|\mathcal{B}_g^* \cap \mathcal{B}_{\sigma(g)}| + |\mathcal{B}_{\sigma(g)}^C \cap \mathcal{B}_g^{*C}|)/(JG)$.

For each setting, we first generate Λ^* once and use them to generate 100 datasets. The averaged results under 100 replication are shown in Table D.1. From the simulation results, we find that our method performs well on the recovery of hierarchical structure.

Table D.1: Simulation results of the recovery of hierarchical factor structure.

J	N	EMC	ACC
20	500	0.94	0.998
	2000	1.00	1.000
40	500	0.88	0.988
	2000	1.00	1.000

E Extraversion Scale Item Key

Table E.1: Extraversion Item Key

Item	Sign	Facet	Item
1	+E1	Friendliness	Make friends easily.
2	+E1	Friendliness	Feel comfortable around people.
3	−E1	Friendliness	Avoid contacts with others.
4	−E1	Friendliness	Keep others at a distance.
5	+E2	Gregariousness	Love large parties.
6	+E2	Gregariousness	Talk to a lot of different people at parties.
7	−E2	Gregariousness	Prefer to be alone.
8	−E2	Gregariousness	Avoid crowds.
9	+E3	Assertiveness	Take charge.
10	+E3	Assertiveness	Try to lead others.
11	+E3	Assertiveness	Take control of things.
12	−E3	Assertiveness	Wait for others to lead the way.
13	+E4	Activity Level	Am always busy.
14	+E4	Activity Level	Am always on the go.
15	+E4	Activity Level	Do a lot in my spare time.
16	−E4	Activity Level	Like to take it easy.
17	+E5	Excitement-Seeking	Love excitement.
18	+E5	Excitement-Seeking	Seek adventure.
19	+E5	Excitement-Seeking	Enjoy being reckless.
20	+E5	Excitement-Seeking	Act wild and crazy.
21	+E6	Cheerfulness	Radiate joy.
22	+E6	Cheerfulness	Have a lot of fun.
23	+E6	Cheerfulness	Love life.
24	+E6	Cheerfulness	Look at the bright side of life.

F Real Data Analysis using Bi-factor Rotation Method

In this section, we present the results of the same data in Section 4 by bi-factor rotation method as a comparison with our proposed method.

Using a candidate set $\mathcal{G} = \{2, \ldots, 12\}$, the BIC procedure of exploratory factor analysis given in Section 3.2 selects eight factors in total, which coincide with the number of factors selected by the BIC procedure of our proposed method. By applying the bi-factor rotation method(Jennrich and Bentler, 2012), we get the

rotation solutions $\widehat{\Lambda}^{oblq}$ in Table F.1 and $\widehat{\Phi}^{oblq}$ in equation (F.1).

Table F.1: Estimated loading matrix $\widehat{\Lambda}^{oblq}$ with seven group factors.

Items	Sign	General	G1	G2	G3	G4	G5	G6	G7
1	+E1	0.86	0.01	-0.06	-0.08	-0.03	-0.04	0.42	-0.08
2	+E1	0.85	0.03	-0.11	0.03	0.06	-0.12	0.07	-0.01
3	−E1	0.91	-0.02	-0.12	-0.01	0.03	-0.09	-0.02	-0.05
4	−E1	0.87	-0.14	-0.04	-0.01	-0.10	-0.14	-0.03	-0.20
5	+E2	0.88	0.69	0.00	0.00	-0.02	0.00	-0.01	0.01
6	+E2	0.92	0.25	0.03	-0.12	0.09	0.05	0.22	-0.03
7	−E2	0.72	-0.06	-0.03	-0.04	-0.08	-0.16	-0.21	-0.12
8	−E2	0.85	0.22	-0.02	-0.05	-0.06	-0.07	-0.29	-0.08
9	+E3	0.52	0.02	0.02	0.00	0.79	0.02	0.04	-0.03
10	+E3	0.52	0.04	0.00	0.00	0.75	-0.03	0.03	0.01
11	+E3	0.44	-0.03	0.00	0.05	0.62	0.04	-0.08	0.01
12	−E3	0.55	-0.09	-0.04	-0.02	0.62	-0.05	-0.06	0.06
13	+E4	0.32	0.05	0.01	0.00	0.02	0.82	-0.02	-0.06
14	+E4	0.51	-0.07	0.02	-0.02	-0.01	0.74	0.06	0.06
15	+E4	0.49	0.02	-0.08	0.15	-0.02	0.51	-0.06	0.14
16	−E4	0.19	-0.14	-0.04	-0.14	0.08	0.37	-0.19	-0.07
17	+E5	0.46	0.09	-0.03	-0.04	0.02	0.02	0.06	0.49
18	+E5	0.53	-0.05	0.02	0.00	0.01	0.03	-0.04	0.62
19	+E5	0.28	0.05	0.48	-0.02	0.02	-0.12	0.00	0.33
20	+E5	0.48	0.00	1.10	0.00	0.00	0.01	0.00	-0.02
21	+E6	0.64	-0.12	0.04	0.26	-0.04	0.01	0.28	-0.01
22	+E6	0.69	0.06	0.11	0.40	-0.01	0.01	0.09	0.08
23	+E6	0.63	0.02	-0.01	0.63	0.03	0.01	-0.05	-0.01
24	+E6	0.58	-0.04	-0.03	0.60	0.00	-0.02	0.01	-0.04

$$\widehat{\Phi}^{oblq} = \begin{pmatrix} 1 & 0 & 0 & 0 & 0 & 0 & 0 & 0 \\ 0 & 1 & 0.19 & -0.14 & -0.17 & -0.15 & 0.10 & 0.05 \\ 0 & 0.19 & 1 & -0.10 & 0.00 & -0.07 & 0.11 & 0.39 \\ 0 & -0.14 & -0.10 & 1 & 0.02 & 0.07 & 0.06 & 0.07 \\ 0 & -0.17 & 0.00 & 0.02 & 1 & 0.21 & -0.07 & 0.11 \\ 0 & -0.15 & -0.07 & 0.07 & 0.21 & 1 & -0.09 & 0.01 \\ 0 & 0.10 & 0.11 & 0.06 & -0.07 & -0.09 & 1 & 0.00 \\ 0 & 0.05 & 0.39 & 0.07 & 0.11 & 0.01 & 0.00 & 1 \end{pmatrix}. \tag{F.1}$$

To help to identify a bi-factor structure from $\widehat{\Lambda}^{oblq}$, all loadings whose absolute value is less than 0.2 are

set to zero, as is done in Jennrich and Bentler (2012). The adjusted loadings are presented in Table F.2. As expected, the loading structure does not conform strictly to a bi-factor model, with four items loading onto three factors.

Table F.2: Estimated bi-factor loading matrix with seven group factors.

Items	Sign	General	G1	G2	G3	G4	G5	G6	G7
1	+E1	0.86	0	0	0	0	0	0.42	0
2	+E1	0.85	0	0	0	0	0	0	0
3	−E1	0.91	0	0	0	0	0	0	0
4	−E1	0.87	0	0	0	0	0	0	-0.20
5	+E2	0.88	0.69	0	0	0	0	0	0
6	+E2	0.92	0.25	0	0	0	0	0.22	0
7	−E2	0.72	0	0	0	0	0	-0.21	0
8	−E2	0.85	0.22	0	0	0	0	-0.29	0
9	+E3	0.52	0	0	0	0.79	0	0	0
10	+E3	0.52	0	0	0	0.75	0	0	0
11	+E3	0.44	0	0	0	0.62	0	0	0
12	−E3	0.55	0	0	0	0.62	0	0	0
13	+E4	0.32	0	0	0	0	0.82	0	0
14	+E4	0.51	0	0	0	0	0.74	0	0
15	+E4	0.49	0	0	0	0	0.51	0	0
16	−E4	0	0	0	0	0	0.37	0	0
17	+E5	0.46	0	0	0	0	0	0	0.49
18	+E5	0.53	0	0	0	0	0	0	0.62
19	+E5	0.28	0	0.48	0	0	0	0	0.33
20	+E5	0.48	0	1.10	0	0	0	0	0
21	+E6	0.64	0	0	0.26	0	0	0.28	0
22	+E6	0.69	0	0	0.40	0	0	0	0
23	+E6	0.63	0	0	0.63	0	0	0	0
24	+E6	0.58	0	0	0.60	0	0	0	0

We now analyze the estimated model in detail. In this result, we have adjusted the sign flip and column swapping to align with the result of the proposed method. All loadings on the general factor are positive, supporting the existence of a general extraversion factor. We interpret the group factors G3, G4, G5 as the Cheerfulness, Assertiveness and Activity Level factors respectively. G2, loaded with the items ”19 Enjoy being reckless” and ”20 Act wild and crazy” is interpreted as the Reckless Excitement-Seeking factor and consistent with the result from our proposed method. G7 is loaded with items ”4 Keep others at a distance”, ”17 Love excitement”, ”18 Seek adventure” and ”19 Enjoy being reckless”. Even though G2 and G7 are loaded with item 19 in common, G7 emphasizes more on the pursuit of meaningful experiences. So we still interpret G7 as the Meaningful Excitement-Seeking factor. Additionally, G2 and G7 are positively correlated,

as is the case in Section 4.

There is a notable difference between the results from the two methods. The result of the ALM method shows the clear presence of a Friendliness factor (G1) and a Gregariousness factor (G6). However, for the bi-factor rotation method, these does not seem to exist a clear Friendliness factor. Both G1 and G6 in the solution of the bi-factor rotation method are related to Gregariousness. Large loadings of the variables designed to measure Friendliness now spread out among several group factors.

Overall, both methods suggest similar (approximate) bi-factor model structures, and the result from the proposed method tends to be neater and more interpretable.

G Technical Proofs

G.1 Proof of Theorem 1

Suppose that $\Lambda\Phi(\Lambda)^\top + \Psi = \Lambda^*\Phi^*(\Lambda^*)^\top + \Psi^*$. Under Condition 1, we have $\Lambda\Phi(\Lambda)^\top = \Lambda^*\Phi^*(\Lambda^*)^\top$. For the simplicity of the notation, we substitute $\Lambda[\mathcal{B}_g^*, \{1, \ldots, G+1\}]$ for $\Lambda[\mathcal{B}_g^*, :]$. The proof consists of three parts:(1) show the bi-factor structure of $\mathcal{B}_{g_1}^*$ is unique, (2) show that combined with some group $g_2 \in \mathcal{H}^*, g_2 \neq g_1$, $\Lambda[\mathcal{B}_{g_1}^*, :]$ is identified up to a sign flip and a group permutation, (3) complete the proof of Theorem 1.

We first consider the equation

$$\Lambda[\mathcal{B}_{g_1}^*, :]\Phi(\Lambda[\mathcal{B}_{g_1}^*, :])^\top = \Lambda^*[\mathcal{B}_{g_1}^*, \{1, 1+g_1\}](\Lambda^*[\mathcal{B}_{g_1}^*, \{1, 1+g_1\}])^\top. \tag{G.1}$$

Since the matrix on the right side of (G.1) has rank 2, there exist 2 possible bi-factor structures for the matrix on the left side of (G.1): (1)$\Lambda[\mathcal{B}_{g_1}^*, \{1, 1+g_1'\}](\Lambda[\mathcal{B}_{g_1}^*, \{1, 1+g_1'\}])^\top = \Lambda^*[\mathcal{B}_{g_1}^*, \{1, 1+g_1\}](\Lambda^*[\mathcal{B}_{g_1}^*, \{1, 1+g_1\}])^\top$ for some $g_1' \in \{1, \ldots, G\}$ and (2) There exists a partition of $\mathcal{B}_{g_1}^* = \mathcal{B}_{g_{1,1}}^* \cup \mathcal{B}_{g_{1,2}}^*$ and $g_1', g_2' \in \{1, \ldots, G\}$ such that

$$\begin{pmatrix} \boldsymbol{\lambda}_1 & \boldsymbol{\lambda}_{g_1'} & \mathbf{0} \\ \boldsymbol{\lambda}_2 & \mathbf{0} & \boldsymbol{\lambda}_{g_2'} \end{pmatrix} \begin{pmatrix} 1 & 0 & 0 \\ 0 & 1 & \phi_{1+g_1',1+g_2'} \\ 0 & \phi_{1+g_1',1+g_2'} & 1 \end{pmatrix} \begin{pmatrix} \boldsymbol{\lambda}_1^\top & \boldsymbol{\lambda}_2^\top \\ \boldsymbol{\lambda}_{g_1'}^\top & \mathbf{0}^\top \\ \mathbf{0}^\top & \boldsymbol{\lambda}_{g_2'}^\top \end{pmatrix} = \begin{pmatrix} \boldsymbol{\lambda}_1^* & \boldsymbol{\lambda}_{g_1}^* \\ \boldsymbol{\lambda}_2^* & \boldsymbol{\lambda}_{g_2}^* \end{pmatrix} \begin{pmatrix} (\boldsymbol{\lambda}_1^*)^\top & (\boldsymbol{\lambda}_2^*)^\top \\ (\boldsymbol{\lambda}_{g_1}^*)^\top & (\boldsymbol{\lambda}_{g_2}^*)^\top \end{pmatrix}, \tag{G.2}$$

where $\boldsymbol{\lambda}_i = \Lambda[\mathcal{B}_{g_{1,i}}^*, \{1\}]$, $\boldsymbol{\lambda}_{g_i'} = \Lambda[\mathcal{B}_{g_{1,i}}^*, \{1+g_i'\}]$, $\boldsymbol{\lambda}_i^* = \Lambda^*[\mathcal{B}_{g_{1,i}}^*, \{1\}]$ and $\boldsymbol{\lambda}_{g_i}^* = \Lambda^*[\mathcal{B}_{g_{1,i}}^*, \{1+g_1\}]$ with $\boldsymbol{\lambda}_{g_i'} \neq \mathbf{0}$ for $i = 1, 2$.

Here we consider the second case. Since the matrix on the right side of (G.2) has rank 2, we must have $(\boldsymbol{\lambda}_i, \boldsymbol{\lambda}_{g_i'})$ has rank 1 for $i = 1, 2$, which leads to the fact that $(\boldsymbol{\lambda}_i^*, \boldsymbol{\lambda}_{g_i}^*)$ has rank 1. However, by

Condition 2, there exists at least one of $(\boldsymbol{\lambda}_1^*, \boldsymbol{\lambda}_{g_1}^*)$ and $(\boldsymbol{\lambda}_2^*, \boldsymbol{\lambda}_{g_2}^*)$ has rank 2. Thus, we must have $\Lambda[\mathcal{B}_{g_1}^*, \{1, 1+g_1'\}](\Lambda[\mathcal{B}_{g_1}^*, \{1, 1+g_1'\}])^\top = \Lambda^*[\mathcal{B}_{g_1}^*, \{1, 1+g_1\}](\Lambda^*[\mathcal{B}_{g_1}^*, \{1, 1+g_1\}])^\top$ for some $g_1' \in \{1, \ldots, G\}$. Without loss of generation, we assume $g_1' = g_1$.

Secondly, there exits some $g_2 \in \mathcal{H}^*$ and $g_2 \neq g_1$ by Condition 2. We consider the $\mathcal{B}_{g_1}^* \cup \mathcal{B}_{g_2}^*$ rows and $\mathcal{B}_{g_1}^* \cup \mathcal{B}_{g_2}^*$ columns of $\Lambda\Phi(\Lambda)^\top$ and $\Lambda^*\Phi^*(\Lambda^*)^\top$. Since the bi-factor structure of the $\mathcal{B}_{g_1}^*$ rows and $\mathcal{B}_{g_1}^*$ columns has already been known, there are two possible bi-factor structures: (1) There exists some $g_2' \in \{1, \ldots, G\}$ such that

$$\begin{pmatrix} \boldsymbol{\lambda}_1 & \boldsymbol{\lambda}_{g_1} & \mathbf{0} \\ \boldsymbol{\lambda}_2 & \mathbf{0} & \boldsymbol{\lambda}_{g_2'} \end{pmatrix} \begin{pmatrix} 1 & 0 & 0 \\ 0 & 1 & \rho_{1,2} \\ 0 & \rho_{1,2} & 1 \end{pmatrix} \begin{pmatrix} \boldsymbol{\lambda}_1^\top & \boldsymbol{\lambda}_2^\top \\ \boldsymbol{\lambda}_{g_1}^\top & \mathbf{0}^\top \\ \mathbf{0}^\top & \boldsymbol{\lambda}_{g_2'}^\top \end{pmatrix} = \begin{pmatrix} \boldsymbol{\lambda}_1^* & \boldsymbol{\lambda}_{g_1}^* & \mathbf{0} \\ \boldsymbol{\lambda}_2^* & \mathbf{0} & \boldsymbol{\lambda}_{g_2}^* \end{pmatrix} \begin{pmatrix} 1 & 0 & 0 \\ 0 & 1 & \rho_{1,2}^* \\ 0 & \rho_{1,2}^* & 1 \end{pmatrix} \begin{pmatrix} (\boldsymbol{\lambda}_1^*)^\top & (\boldsymbol{\lambda}_2^*)^\top \\ (\boldsymbol{\lambda}_{g_1}^*)^\top & \mathbf{0}^\top \\ \mathbf{0}^\top & (\boldsymbol{\lambda}_{g_2}^*)^\top \end{pmatrix}, \tag{G.3}$$

where $\boldsymbol{\lambda}_i = \Lambda[\mathcal{B}_{g_i}^*, \{1\}]$, $\boldsymbol{\lambda}_{g_i'} = \Lambda[\mathcal{B}_{g_i}^*, \{1 + g_i'\}]$, $\boldsymbol{\lambda}_i^* = \Lambda^*[\mathcal{B}_{g_i}^*, \{1\}]$ and $\boldsymbol{\lambda}_{g_i}^* = \Lambda^*[\mathcal{B}_{g_i}^*, \{1 + g_1\}]$ for $i = 1, 2$. $\rho_{1,2} = \phi_{1+g_1', 1+g_2'}$ and $\rho_{1,2}^* = \phi_{1+g_1, 1+g_2}^*$.

(2) There exists a partition of $\mathcal{B}_{g_2}^* = \mathcal{B}_{g_2,1}^* \cup \mathcal{B}_{g_2,2}^*$ and $g_2' \in \{1, \ldots, G\}$ such that

$$\begin{aligned} &\begin{pmatrix} \boldsymbol{\lambda}_1 & \boldsymbol{\lambda}_{g_1} & \mathbf{0} \\ \boldsymbol{\lambda}_{2,1} & \boldsymbol{\lambda}_{2,g_1} & \mathbf{0} \\ \boldsymbol{\lambda}_{2,2} & \mathbf{0} & \boldsymbol{\lambda}_{2,g_2'} \end{pmatrix} \begin{pmatrix} 1 & 0 & 0 \\ 0 & 1 & \rho_{1,2} \\ 0 & \rho_{1,2} & 1 \end{pmatrix} \begin{pmatrix} \boldsymbol{\lambda}_1^\top & \boldsymbol{\lambda}_{2,1}^\top & \boldsymbol{\lambda}_{2,2}^\top \\ \boldsymbol{\lambda}_{g_1}^\top & \boldsymbol{\lambda}_{2,g_1}^\top & \mathbf{0}^\top \\ \mathbf{0}^\top & \mathbf{0}^\top & \boldsymbol{\lambda}_{2,g_2'}^\top \end{pmatrix} \\ = &\begin{pmatrix} \boldsymbol{\lambda}_1^* & \boldsymbol{\lambda}_{g_1}^* & \mathbf{0} \\ \boldsymbol{\lambda}_{2,1}^* & \mathbf{0} & \boldsymbol{\lambda}_{g_2,1}^* \\ \boldsymbol{\lambda}_{2,2}^* & \mathbf{0} & \boldsymbol{\lambda}_{g_2,2}^* \end{pmatrix} \begin{pmatrix} 1 & 0 & 0 \\ 0 & 1 & \rho_{1,2}^* \\ 0 & \rho_{1,2}^* & 1 \end{pmatrix} \begin{pmatrix} (\boldsymbol{\lambda}_1^*)^\top & (\boldsymbol{\lambda}_{2,1}^*)^\top & (\boldsymbol{\lambda}_{2,2}^*)^\top \\ (\boldsymbol{\lambda}_{g_1}^*)^\top & \mathbf{0}^\top & \mathbf{0}^\top \\ \mathbf{0}^\top & (\boldsymbol{\lambda}_{g_2,1}^*)^\top & (\boldsymbol{\lambda}_{g_2,2}^*)^\top \end{pmatrix}, \end{aligned} \tag{G.4}$$

where $\boldsymbol{\lambda}_1 = \Lambda[\mathcal{B}_{g_1}^*, \{1\}]$, $\boldsymbol{\lambda}_{g_1} = \Lambda[\mathcal{B}_{g_1}^*, \{1 + g_1\}]$, $\boldsymbol{\lambda}_1^* = \Lambda^*[\mathcal{B}_{g_1}^*, \{1\}]$, $\boldsymbol{\lambda}_{g_1}^* = \Lambda^*[\mathcal{B}_{g_1}^*, \{1 + g_1\}]$, $\boldsymbol{\lambda}_{2,i} = \Lambda[\mathcal{B}_{g_2,i}^*, \{1\}]$, $\boldsymbol{\lambda}_{2,i}^* = \Lambda[\mathcal{B}_{g_2,i}^*, \{1\}]$, $\boldsymbol{\lambda}_{g_2,i}^* = \Lambda[\mathcal{B}_{g_2,i}^*, \{1 + g_2\}]$ for $i = 1, 2$, $\boldsymbol{\lambda}_{2,g_1} = \Lambda[\mathcal{B}_{g_2,1}^*, \{1 + g_1\}]$, $\boldsymbol{\lambda}_{2,g_2'} = \Lambda[\mathcal{B}_{g_2,2}^*, \{1 + g_2'\}]$, $\rho_{1,2} = \phi_{1+g_1, 1+g_2'}$ and $\rho_{1,2}^* = \phi_{1+g_1, 1+g_2}^*$.

For the second case in (G.4), there exists some α such that $\boldsymbol{\lambda}_1 = \cos\alpha\boldsymbol{\lambda}_1^* - \sin\alpha\boldsymbol{\lambda}_{g_1}^*$ and $\boldsymbol{\lambda}_{g_1} = \sin\alpha\boldsymbol{\lambda}_1^* + \cos\alpha\boldsymbol{\lambda}_{g_1}^*$. Since the $\mathcal{B}_{g_2}^*$ rows and the $\mathcal{B}_{g_2}^*$ columns of $\Lambda\Phi(\Lambda)^\top$ and $\Lambda^*\Phi^*(\Lambda^*)^\top$ have rank 2, under the bi-factor structure of the second case, we have that $(\boldsymbol{\lambda}_{2,1}, \boldsymbol{\lambda}_{2,g_1}, \boldsymbol{\lambda}_{2,1}^*, \boldsymbol{\lambda}_{g_2,1}^*)$ has rank 1 and $(\boldsymbol{\lambda}_{2,2}, \boldsymbol{\lambda}_{2,g_2'}, \boldsymbol{\lambda}_{2,2}^*, \boldsymbol{\lambda}_{g_2,2}^*)$ has rank 1. Noticing that $\boldsymbol{\lambda}_{g_2,1}^* \neq \mathbf{0}$, we assume that $\lambda_{2,1}^* = k_1\boldsymbol{\lambda}_{g_2,1}^*$, $\boldsymbol{\lambda}_{2,1} = k_2\boldsymbol{\lambda}_{g_2,1}^*$ and $\boldsymbol{\lambda}_{2,g_1} = k_3\boldsymbol{\lambda}_{g_2,1}^*$. For the $\mathcal{B}_{g_2,1}^*$ rows and the $\mathcal{B}_{g_2,1}^*$ columns of (G.4), we have $1 + k_1^2 = k_2^2 + k_3^2$. For the $\mathcal{B}_{g_1}^*$ rows and the $\mathcal{B}_{g_2,1}^*$

columns of (G.4), we have

$$
\begin{aligned}
&\boldsymbol{\lambda}_1^*(\boldsymbol{\lambda}_{2,1}^*)^\top + \rho_{1,2}^*\boldsymbol{\lambda}_{g_1}^*(\boldsymbol{\lambda}_{g_2,1}^*)^\top \\
=&\boldsymbol{\lambda}_1(\boldsymbol{\lambda}_{2,1})^\top + \boldsymbol{\lambda}_{g_1}(\boldsymbol{\lambda}_{2,g_1})^\top \\
=&k_2(\cos\alpha\boldsymbol{\lambda}_1^* - \sin\alpha\boldsymbol{\lambda}_{g_1}^*)(\boldsymbol{\lambda}_{g_2,1}^*)^\top + k_3(\sin\alpha\boldsymbol{\lambda}_1^* + \cos\alpha\boldsymbol{\lambda}_{g_1}^*)(\boldsymbol{\lambda}_{g_2,1}^*)^\top \\
=&(k_2\cos\alpha + k_3\sin\alpha)\boldsymbol{\lambda}_1^*(\boldsymbol{\lambda}_{g_2,1}^*)^\top + (k_3\cos\alpha - k_2\sin\alpha)\boldsymbol{\lambda}_{g_1}^*(\boldsymbol{\lambda}_{g_2,1}^*)^\top.
\end{aligned}
$$

Then, we have $k_1 = k_2\cos\alpha + k_3\sin\alpha$ and $\rho_{1,2}^* = k_3\cos\alpha - k_2\sin\alpha$, which leads to $k_1^2 + (\rho_{1,2}^*)^2 = k_2^2 + k_3^2$. Combined with $1 + k_1^2 = k_2^2 + k_3^2$, we have $|\rho_{1,2}^*| = 1$, which contradicts to the fact that Φ^* is positive definite. Thus, only the first case is allowed. Without loss of generation, we assume $g_2' = g_2$.

For the first case in (G.3), there exists some α, β such that $\boldsymbol{\lambda}_1 = \cos\alpha\boldsymbol{\lambda}_1^* - \sin\alpha\boldsymbol{\lambda}_{g_1}^*$, $\boldsymbol{\lambda}_{g_1} = \sin\alpha\boldsymbol{\lambda}_1^* + \cos\alpha\boldsymbol{\lambda}_{g_1}^*$, $\boldsymbol{\lambda}_2 = \cos\beta\boldsymbol{\lambda}_2^* - \sin\beta\boldsymbol{\lambda}_{g_2}^*$ and $\boldsymbol{\lambda}_{g_2} = \sin\beta\boldsymbol{\lambda}_2^* + \cos\beta\boldsymbol{\lambda}_{g_2}^*$. We then have the following equation

$$
\begin{pmatrix} \cos\alpha & \sin\alpha \\ -\sin\alpha & \cos\alpha \end{pmatrix}
\begin{pmatrix} 1 & 0 \\ 0 & \rho_{12} \end{pmatrix}
\begin{pmatrix} \cos\beta & -\sin\beta \\ \sin\beta & \cos\beta \end{pmatrix}
=
\begin{pmatrix} 1 & 0 \\ 0 & \rho_{12}^* \end{pmatrix},
$$

which leads to $1 = \cos\alpha\cos\beta + \rho_{12}\sin\alpha\sin\beta$. Since $|\rho_{12}| < 1$, we have $\cos\alpha\cos\beta = 1$ and $\sin\alpha\sin\beta = 0$. Without loss of generation, we assume $\cos\alpha = \cos\beta = 1$. Then we have $\boldsymbol{\lambda}_1 = \boldsymbol{\lambda}_1^*$, $\boldsymbol{\lambda}_{g_1} = \boldsymbol{\lambda}_{g_1}^*$, $\boldsymbol{\lambda}_2 = \boldsymbol{\lambda}_2^*$, $\boldsymbol{\lambda}_{g_2} = \boldsymbol{\lambda}_{g_2}^*$ and $\rho_{12} = \rho_{12}^*$.

For any group $g_3 \neq g_1, g_2$, we consider the $\mathcal{B}_{g1}^* \cup \mathcal{B}_{g3}^*$ rows and $\mathcal{B}_{g1}^* \cup \mathcal{B}_{g3}^*$ columns of $\Lambda\Phi(\Lambda)^\top$ and $\Lambda^*\Phi^*(\Lambda^*)^\top$. Similar to the the proof of g_2, there exists only one possible bi-factor structure : For some $g_3' \in \{1, \ldots, G\}$, we have

$$
\begin{pmatrix} \boldsymbol{\lambda}_1 & \boldsymbol{\lambda}_{g_1'} & \mathbf{0} \\ \boldsymbol{\lambda}_3 & \mathbf{0} & \boldsymbol{\lambda}_{g_3'} \end{pmatrix}
\begin{pmatrix} 1 & 0 & 0 \\ 0 & 1 & \rho_{1,3} \\ 0 & \rho_{1,3} & 1 \end{pmatrix}
\begin{pmatrix} \boldsymbol{\lambda}_1^\top & \boldsymbol{\lambda}_3^\top \\ \boldsymbol{\lambda}_{g_1'}^\top & \mathbf{0}^\top \\ \mathbf{0}^\top & \boldsymbol{\lambda}_{g_3'}^\top \end{pmatrix}
=
\begin{pmatrix} \boldsymbol{\lambda}_1^* & \boldsymbol{\lambda}_{g_1}^* & \mathbf{0} \\ \boldsymbol{\lambda}_3^* & \mathbf{0} & \boldsymbol{\lambda}_{g_3}^* \end{pmatrix}
\begin{pmatrix} 1 & 0 & 0 \\ 0 & 1 & \rho_{1,3}^* \\ 0 & \rho_{1,3}^* & 1 \end{pmatrix}
\begin{pmatrix} (\boldsymbol{\lambda}_1^*)^\top & (\boldsymbol{\lambda}_3^*)^\top \\ (\boldsymbol{\lambda}_{g_1}^*)^\top & \mathbf{0}^\top \\ \mathbf{0}^\top & (\boldsymbol{\lambda}_{g_3}^*)^\top \end{pmatrix},
$$

where $\boldsymbol{\lambda}_i = \Lambda[\mathcal{B}_{g_i}^*, \{1\}]$, $\boldsymbol{\lambda}_{g_i'} = \Lambda[\mathcal{B}_{g_i}^*, \{1 + g_i'\}]$, $\boldsymbol{\lambda}_i^* = \Lambda^*[\mathcal{B}_{g_i}^*, \{1\}]$ and $\boldsymbol{\lambda}_{g_i}^* = \Lambda^*[\mathcal{B}_{g_i}^*, \{1 + g_1\}]$ for $i = 1, 3$. $\rho_{1,3} = \phi_{1+g_1', 1+g_3'}$ and $\rho_{1,3}^* = \phi_{1+g_1, 1+g_3}^*$. We then have

$$
\begin{aligned}
\boldsymbol{\lambda}_1^*(\boldsymbol{\lambda}_3^*)^\top + \rho_{1,3}^*\boldsymbol{\lambda}_{g_1}^*(\boldsymbol{\lambda}_{g_3}^*)^\top &= \boldsymbol{\lambda}_1(\boldsymbol{\lambda}_3)^\top + \rho_{1,3}\boldsymbol{\lambda}_{g_1}(\boldsymbol{\lambda}_{g_3'})^\top \\
\boldsymbol{\lambda}_3^*(\boldsymbol{\lambda}_3^*)^\top + \boldsymbol{\lambda}_{g_3}^*(\boldsymbol{\lambda}_{g_3}^*)^\top &= \boldsymbol{\lambda}_3(\boldsymbol{\lambda}_3)^\top + \boldsymbol{\lambda}_{g_3}(\boldsymbol{\lambda}_{g_3})^\top.
\end{aligned}
$$

Since we have proved $\boldsymbol{\lambda}_1^* = \boldsymbol{\lambda}_1$ and $\boldsymbol{\lambda}_{g_1}^* = \boldsymbol{\lambda}_{g_1}$, we then have $\boldsymbol{\lambda}_3^* = \boldsymbol{\lambda}_3$, $\boldsymbol{\lambda}_{g_3}^* = \boldsymbol{\lambda}_{g_3'}$, $\rho_{1,3}^* = \rho_{1,3}$ or $\boldsymbol{\lambda}_{g_3}^* = -\boldsymbol{\lambda}_{g_3'}$,

$\rho^*_{1,3} = -\rho_{1,3}$.

Now, since Λ has G group factors, according to the previous proof, each variable belonging to $\mathcal{B}^*_i$ loads on a unique group factor according to Λ and the loadings of the general factor and the group factors are determined up to a sign flip for $i = 1, \ldots, G$. Thus, there exist a diagonal sign-flip matrix $D \in \mathcal{D}$ and a permutation matrix $P \in \mathcal{P}$ such that $\Lambda = \Lambda^* PD$. It is straightforward to further check that $\Phi = DP^\top \Phi^* PD$. Thus, the proof is completed.

G.2 Identifiability of Estimated Bi-factor Structure in Real Data Example

For any matrix A, we use $\mathrm{rank}(A)$ to denote the rank of A. The following condition is a necessary condition for the identifiability of the extended bi-factor model under a known bi-factor structure, as proposed in Theorem 3 of Fang et al. (2021).

Condition 4. $|\mathcal{B}_g| \geqslant 2$ *for all* $g = 1, \ldots, G$.

We then propose the following condition for the identifiability of parameters when the true bi-factor structure is the same as the estimated structure in Section 4.

Condition 5. *For any* $m \times n$ *dimensional submatrix of* $\Phi[\{2, \ldots, 1+G\}, \{2, \ldots, 1+G\}]$, $1 \leqslant m, n \leqslant G$, *it's rank is* $\min(m, n)$.

Condition 6. *For any* g *such that* $|\mathcal{B}^*_g| \geqslant 3$, *any 2 rows of* $\Lambda^*[\mathcal{B}^*_g, \{1, 1+g\}]$ *are linearly independent.*

Remark 4. *Condition 5 restricts that the correlation matrix of group factors does not degenerate. In Theorem 2, we restrict the parametric space of* Φ *to the space satisfying Condition 5. We note that* $\widehat{\Lambda}$ *in Section 4 satisfies Condition 5. Condition 6 is easy to check in practice and* $\widehat{\Lambda}$ *in Section 4 satisfies Condition 6.*

Theorem 2. *Suppose the true bi-factor structure follows* $\widehat{\Lambda}$ *in Section 4. Let* Λ^*, Φ^* *and* Ψ^* *be the true parameters such that Conditions 4 -6 are satisfied. For any parameters* Λ, Φ *and* Ψ *that satisfy Conditions 4 and 5 and* $\Lambda^*\Phi^*(\Lambda^*)^\top + \Psi^* = \Lambda\Phi(\Lambda)^\top + \Psi$, *there exists a diagonal sign-flip matrix* $D \in \mathcal{D}$ *and a permutation matrix* $P \in \mathcal{P}$ *such that* $\Lambda = \Lambda^* PD$, $\Phi = DP^\top \Phi^* PD$ *and* $\Psi^* = \Psi$.

Proof of Theorem 2 : Without loss of generation, we assume that $|\mathcal{B}^*_1| = |\mathcal{B}^*_2| = 5$, $|\mathcal{B}^*_3| = |\mathcal{B}^*_4| = 4$ and $|\mathcal{B}^*_5| = |\mathcal{B}^*_6| = |\mathcal{B}^*_7| = 2$. Suppose that there exists Λ, Φ and Ψ and $\Sigma = \Lambda\Phi\Lambda^\top + \Psi$ such that $\Sigma = \Sigma^*$. The proof consists of two parts: (1) Show that $\Lambda[\cup_{i=1}^4 \mathcal{B}^*_i, :]$ and $\Lambda^*[\cup_{i=1}^4 \mathcal{B}^*_i, :]$ has the same bi-factor structure. Without loss of generality, we further assume that $\Lambda[\mathcal{B}^*_i, \{1+i\}] \neq \mathbf{0}$ for $i = 1, \ldots, 4$. We show that there exists some 5×5 sign flip matrix $\widetilde{D}$ such that $\Lambda[\cup_{i=1}^4 \mathcal{B}^*_i, \{1, \ldots, 5\}] = \Lambda^*[\cup_{i=1}^4 \mathcal{B}^*_i, \{1, \ldots, 5\}]\widetilde{D}$,

$\Phi[\{1,\ldots,5\},\{1,\ldots,5\}] = \widetilde{D}\Phi^*[\{1,\ldots,5\},\{1,\ldots,5\}]\widetilde{D}$ and $\psi_j = \psi_j^*$ for $j \in \cup_{i=1}^4 \mathcal{B}_i^*$. (2) Show that Λ and Λ^* have the same bi-factor structure for the rest of the variables and complete the proof.

We now prove the first part. Let $\mathcal{F}_i = \{g : \Lambda[\mathcal{B}_i^*, \{1+g\}] \neq \mathbf{0}\} \cup \{1\}$ be the set of factors such that the variables belonging to $\mathcal{B}_i^*$ load on these factors for $i = 1,\ldots,4$. We note that $|\mathcal{F}_i| \geqslant 2$ for $i = 1,\ldots,4$. When $|\mathcal{F}_i| = 2$, all variables that belong to $\mathcal{B}_i^*$ load on the same group factor. We claim that

$$\operatorname{rank}(\Lambda[\mathcal{B}_i^*, \mathcal{F}_i]) = |\mathcal{F}_i| \ \text{ if } \ |\mathcal{F}_i| \leqslant |\mathcal{B}_i^*| \ \text{ for } \ i = 1,\ldots,4. \tag{G.5}$$

If $|\mathcal{F}_i| \leqslant |\mathcal{B}_i^*|$, there exists some $g_i \in \mathcal{F}_i$, $g_i \neq 1$ and $j_{g_i}, j'_{g_i} \in \mathcal{B}_i^*$ such that $\lambda_{j_{g_i},1+g_i} \neq 0$ and $\lambda_{j'_{g_i},1+g_i} \neq 0$. For $1 \leqslant i' \leqslant 4$, $i' \neq i$, consider the equation $\Sigma[\{j_{g_i}, j'_{g_i}\}, \mathcal{B}_{i'}^*] = \Sigma^*[\{j_{g_i}, j'_{g_i}\}, \mathcal{B}_{i'}^*]$, which is equivalent to

$$\begin{aligned} &\Lambda[\{j_{g_i}, j'_{g_i}\}, \{1, 1+g_i\}]\Phi[\{1, 1+g_i\}, \mathcal{F}_{i'}](\Lambda[\mathcal{B}_{i'}^*, \mathcal{F}_{i'}])^\top \\ =&\Lambda^*[\{j_{g_i}, j'_{g_i}\}, \{1, 1+i\}]\Phi^*[\{1, 1+i\}, \{1, 1+i'\}](\Lambda^*[\mathcal{B}_{i'}^*, \{1, 1+i'\}])^\top. \end{aligned} \tag{G.6}$$

Noticing that by Condition 5 and 6 hold for Φ^* and Λ^*,

$$\Lambda^*[\{j_{g_i}, j'_{g_i}\}, \{1, 1+i\}]\Phi^*[\{1, 1+i\}, \{1, 1+i'\}](\Lambda^*[\mathcal{B}_{i'}^*, \{1, 1+i'\}])^\top$$

has rank 2. Thus, $\Lambda[\{j_{g_i}, j'_{g_i}\}, \{1, 1+g_i\}]$ should have rank 2. Otherwise, $\Lambda[\{j_i, j'_i\}, \{1, 1+g_i\}]\Phi[\{1, 1+g_i\}, \mathcal{F}_{i'}](\Lambda[\mathcal{B}_{i'}^*, \mathcal{F}_{i'}])^\top$ has at most rank 1, which contradicts (G.6). Then, since for each $g'_i \in \mathcal{F}_i$, $g'_i \neq 1$, there exists some $j_{g'_i}$ such that $\lambda_{j_{g'_i}, g'_i} \neq 0$, it is easy to check that (G.5) holds.

Then, consider the equation $\Sigma[\mathcal{B}_i^*, \mathcal{B}_{i'}^*] = \Sigma^*[\mathcal{B}_i^*, \mathcal{B}_{i'}^*]$ for $1 \leqslant i \neq i' \leqslant 4$, which is equivalent to

$$\Lambda[\mathcal{B}_i^*, \mathcal{F}_i]\Phi[\mathcal{F}_i, \mathcal{F}_{i'}](\Lambda[\mathcal{B}_{i'}^*, \mathcal{F}_{i'}])^\top = \Lambda^*[\mathcal{B}_i^*, \{1, 1+i\}]\Phi[\{1, 1+i\}, \{1, 1+i'\}](\Lambda[\mathcal{B}_{i'}^*, \{1, 1+i'\}])^\top.$$

With the same argument, $\Lambda^*[\mathcal{B}_i^*, \{1, 1+i\}]\Phi[\{1, 1+i\}, \{1, 1+i'\}](\Lambda[\mathcal{B}_{i'}^*, \{1, 1+i'\}])^\top$ has rank 2. By Sylvester's rank inequality, we have

$$\begin{aligned} &\operatorname{rank}(\Lambda[\mathcal{B}_i^*, \mathcal{F}_i]\Phi[\mathcal{F}_i, \mathcal{F}_{i'}](\Lambda[\mathcal{B}_{i'}^*, \mathcal{F}_{i'}])^\top) \\ \geqslant&\operatorname{rank}((\Lambda[\mathcal{B}_i^*, \mathcal{F}_i]) + \operatorname{rank}(\Phi[\mathcal{F}_i, \mathcal{F}_{i'}]) + \operatorname{rank}(\Lambda[\mathcal{B}_{i'}^*, \mathcal{F}_{i'}]) - |\mathcal{F}_i| - |\mathcal{F}_{i'}|. \end{aligned} \tag{G.7}$$

We consider the following case

1. $|\mathcal{F}_i| \leqslant |\mathcal{B}_i^*|$ for all $1 \leqslant i \leqslant 4$. In this case, according to claim (G.5) and Condition 5, inequality (G.7)

leads to

$$\mathrm{rank}(\Lambda[\mathcal{B}_i^*, \mathcal{F}_i]\Phi[\mathcal{F}_i, \mathcal{F}_{i'}](\Lambda[\mathcal{B}_{i'}^*, \mathcal{F}_{i'}])^\top) \geqslant \min(|\mathcal{F}_i|, |\mathcal{F}_{i'}|),$$

for any $1 \leqslant i \neq i' \leqslant 4$. We then have $\min(|\mathcal{F}_i|, |\mathcal{F}_{i'}|) = 2$. By applying this argument to all pairs (i, i'), $1 \leqslant i < i' \leqslant 4$, we conclude that there exists at most one $\mathcal{F}_i$ such that $|\mathcal{F}_i| \geqslant 3$ for $1 \leqslant i \leqslant 4$.

If there exists some i such that $|\mathcal{F}_i| \geqslant 3$ for $i = 1, \dots, 4$. Without loss of generality, we assume $|\mathcal{F}_1| \geqslant 3$ and $|\mathcal{F}_i| = 2$ for $i = 2, 3, 4$. We claim that $|\mathcal{F}_2 \cup \mathcal{F}_3| = 3$, in other words, the variables belonging to $\mathcal{B}_2^*$ and $\mathcal{B}_3^*$ load on different factors. Otherwise, $|\mathcal{F}_2 \cup \mathcal{F}_3| = 2$. Consider the equation

$$\Sigma[\mathcal{B}_2^* \cup \mathcal{B}_3^*, \mathcal{B}_2^* \cup \mathcal{B}_3^*] = \Sigma^*[\mathcal{B}_2^* \cup \mathcal{B}_3^*, \mathcal{B}_2^* \cup \mathcal{B}_3^*],$$

which is equivalent to

$$\begin{aligned} &\Lambda[\mathcal{B}_2^* \cup \mathcal{B}_3^*, \mathcal{F}_2 \cup \mathcal{F}_3]\Phi[\mathcal{F}_2 \cup \mathcal{F}_3, \mathcal{F}_2 \cup \mathcal{F}_3](\Lambda[\mathcal{B}_2^* \cup \mathcal{B}_3^*, \mathcal{F}_2 \cup \mathcal{F}_3])^\top + \Psi[\mathcal{B}_2^* \cup \mathcal{B}_3^*, \mathcal{B}_2^* \cup \mathcal{B}_3^*] \\ =&\Lambda^*[\mathcal{B}_2^* \cup \mathcal{B}_3^*, \{1,3,4\}]\Phi^*[\{1,3,4\}, \{1,3,4\}](\Lambda^*[\mathcal{B}_2^* \cup \mathcal{B}_3^*, \{1,3,4\}])^\top + \Psi^*[\mathcal{B}_2^* \cup \mathcal{B}_3^*, \mathcal{B}_2^* \cup \mathcal{B}_3^*]. \end{aligned} \tag{G.8}$$

Since Λ^* satisfies Condition 6, noticing that $|\mathcal{B}_2^*| \geqslant 4$ and $|\mathcal{B}_3^*| \geqslant 4$, it is easy to check that the matrix $\Lambda^*[\mathcal{B}_2^* \cup \mathcal{B}_3^*, \{1,3,4\}]$ satisfies the condition for Theorem 5.1 of Anderson and Rubin (1956), that is, if any row of $\Lambda^*[\mathcal{B}_2^* \cup \mathcal{B}_3^*, \{1,3,4\}]$ is deleted, there still remains two disjoint submatrices of $\Lambda^*[\mathcal{B}_2^* \cup \mathcal{B}_3^*, \{1,3,4\}]$ with rank 3. By applying Theorem 5.1 of Anderson and Rubin (1956), we have $\Psi[\mathcal{B}_2^* \cup \mathcal{B}_3^*, \mathcal{B}_2^* \cup \mathcal{B}_3^*] = \Psi^*[\mathcal{B}_2^* \cup \mathcal{B}_3^*, \mathcal{B}_2^* \cup \mathcal{B}_3^*]$. Thus, we further have

$$\begin{aligned} &\Lambda[\mathcal{B}_2^* \cup \mathcal{B}_3^*, \mathcal{F}_2 \cup \mathcal{F}_3]\Phi[\mathcal{F}_2 \cup \mathcal{F}_3, \mathcal{F}_2 \cup \mathcal{F}_3](\Lambda[\mathcal{B}_2^* \cup \mathcal{B}_3^*, \mathcal{F}_2 \cup \mathcal{F}_3])^\top \\ =&\Lambda^*[\mathcal{B}_2^* \cup \mathcal{B}_3^*, \{1,3,4\}]\Phi^*[\{1,3,4\}, \{1,3,4\}](\Lambda^*[\mathcal{B}_2^* \cup \mathcal{B}_3^*, \{1,3,4\}])^\top. \end{aligned} \tag{G.9}$$

If $|\mathcal{F}_2 \cup \mathcal{F}_3| = 2$, then the rank of the matrix in the first line of (G.9) is 2, which contradicts the fact that the rank of the matrix in the second line of (G.9) is 3. Thus, $|\mathcal{F}_2 \cup \mathcal{F}_3| = 3$. We note that with a similar argument used in (G.8) and (G.9), we also have $|\mathcal{F}_2 \cup \mathcal{F}_4| = 3$, $|\mathcal{F}_3 \cup \mathcal{F}_4| = 3$ and $|\cup_{i=2,3,4} \mathcal{F}_i| = 4$. Then, consider the equation

$$\Sigma[\mathcal{B}_1^*, \mathcal{B}_2^* \cup \mathcal{B}_3^*] = \Sigma^*[\mathcal{B}_1^*, \mathcal{B}_2^* \cup \mathcal{B}_3^*],$$

which is equivalent to

$$\begin{aligned}&\Lambda[\mathcal{B}_1^*,\mathcal{F}_1]\Phi[\mathcal{F}_1,\mathcal{F}_2\cup\mathcal{F}_3](\Lambda[\mathcal{B}_2^*\cup\mathcal{B}_3^*,\mathcal{F}_2\cup\mathcal{F}_3)^\top\\ =&\Lambda^*[\mathcal{B}_1^*,\{1,2\}]\Phi^*[\{1,2\},\{1,3,4\}](\Lambda^*[\mathcal{B}_2^*\cup\mathcal{B}_3^*,\{1,3,4\}])^\top.\end{aligned}\tag{G.10}$$

We note that the rank of the matrix in the second line of (G.10) is 2. According to Sylvester's rank inequality

$$\begin{aligned}&\operatorname{rank}(\Lambda[\mathcal{B}_1^*,\mathcal{F}_1]\Phi[\mathcal{F}_1,\mathcal{F}_2\cup\mathcal{F}_3](\Lambda[\mathcal{B}_2^*\cup\mathcal{B}_3^*,\mathcal{F}_2\cup\mathcal{F}_3)^\top)\\ \geqslant&\operatorname{rank}(\Lambda[\mathcal{B}_1^*,\mathcal{F}_1])+\operatorname{rank}(\Phi[\mathcal{F}_1,\mathcal{F}_2\cup\mathcal{F}_3])+\operatorname{rank}(\Lambda[\mathcal{B}_2^*\cup\mathcal{B}_3^*,\mathcal{F}_2\cup\mathcal{F}_3])-|\mathcal{F}_1|-3\\ =&|\mathcal{F}_1|+\min(|\mathcal{F}_1|,3)+3-|\mathcal{F}_1|-3\\ =&3,\end{aligned}$$

which contradicts (G.10).

Thus, in the case, $|\mathcal{F}_i|=2$ for $i=1,\ldots,4$. Consider the equation

$$\Sigma[\cup_{i=1,\ldots,4}\mathcal{B}_i^*,\cup_{i=1,\ldots,4}\mathcal{B}_i^*]=\Sigma^*[\cup_{i=1,\ldots,4}\mathcal{B}_i^*,\cup_{i=1,\ldots,4}\mathcal{B}_i^*].\tag{G.11}$$

By the similar argument discussed in (G.8), (G.9) and further applying Theorem 1 to (G.11), we conclude that in this case, $\Lambda[\cup_{i=1}^4\mathcal{B}_i^*,:]$ and $\Lambda^*[\cup_{i=1}^4\mathcal{B}_i^*,:]$ has the same bi-factor structure. Without loss of generality, we further assume that $\mathcal{F}_i=\{1,1+i\}$ for $i=1,\ldots,4$. Then, there exists some 5×5 sign flip matrix $\widetilde{D}$ such that $\Lambda[\cup_{i=1}^4\mathcal{B}_i^*,\{1,\ldots,5\}]=\Lambda^*[\cup_{i=1}^4\mathcal{B}_i^*,\{1,\ldots,5\}]\widetilde{D}$, $\Phi[\{1,\ldots,5\},\{1,\ldots,5\}]=\widetilde{D}\Phi^*[\{1,\ldots,5\},\{1,\ldots,5\}]\widetilde{D}$ and $\psi_j=\psi_j^*$ for $j\in\cup_{i=1}^4\mathcal{B}_i^*$.

2. There exists some $1\leqslant i\leqslant 4$ such that $|\mathcal{F}_i|=1+|\mathcal{B}_i^*|\geqslant 5$. In this case, according to (G.7)

$$\operatorname{rank}(\Lambda[\mathcal{B}_i^*,\mathcal{F}_i]\Phi[\mathcal{F}_i,\mathcal{F}_{i'}](\Lambda[\mathcal{B}_{i'}^*,\mathcal{F}_{i'}])^\top)\geqslant 3\ \text{ if }\ |\mathcal{F}_{i'}|\geqslant 4.$$

Thus, $|\mathcal{F}_{i'}|\leqslant 3<|\mathcal{B}_{i'}^*|$ for all $1\leqslant i'\leqslant 4$, $i'\neq i$. Without loss of generality, let $i=1$. For $i'=2,3,4$, by the same argument in case 1, we have $\mathcal{F}_2=\{1,1+g_2\}$, $\mathcal{F}_3=\{1,1+g_3\}$ and $\mathcal{F}_4=\{1,1+g_4\}$ for different g_2,g_3 and g_4. Moreover, $\operatorname{rank}(\Lambda[\cup_{i=2,3,4}\mathcal{B}_i^*,\cup_{i=2,3,4}\mathcal{F}_i])=4$.

Then, consider the equation $\Sigma[\mathcal{B}_1^*,\cup_{i=2,3,4}\mathcal{B}_i^*]=\Sigma^*[\mathcal{B}_1^*,\cup_{i=2,3,4}\mathcal{B}_i^*]$, which is equivalent to

$$\begin{aligned}&\Lambda[\mathcal{B}_1^*,\mathcal{F}_1]\Phi[\mathcal{F}_1,\cup_{i=2,3,4}\mathcal{F}_i](\Lambda[\cup_{i=2,3,4}\mathcal{B}_i^*,\cup_{i=2,3,4}\mathcal{F}_i])^\top\\ =&\Lambda^*[\mathcal{B}_1^*,\{1,2\}]\Phi^*[\{1,2\},\{1,3,4,5\}](\Lambda[\cup_{i=2,3,4}\mathcal{B}_i^*,\{1,3,4,5\}])^\top\end{aligned}\tag{G.12}$$

It is straightforward that $\Lambda^*[\mathcal{B}_1^*, \{1,2\}]\Phi^*[\{1,2\},\{1,3,4,5\}](\Lambda[\cup_{i=2,3,4}\mathcal{B}_i^*, \{1,3,4,5\}])^\top$ has rank 2 according to Condition 5 and 6. While, since $|\cup_{i=2,3,4}\mathcal{F}_i| = 4 < |\mathcal{F}_1|$, according to Sylvester's rank inequality,

$$
\begin{aligned}
&\operatorname{rank}(\Lambda[\mathcal{B}_1^*, \mathcal{F}_1]\Phi[\mathcal{F}_1, \cup_{i=2,3,4}\mathcal{F}_i](\Lambda[\cup_{i=2,3,4}\mathcal{B}_i^*, \cup_{i=2,3,4}\mathcal{F}_i])^\top) \\
\geqslant&\operatorname{rank}(\Lambda[\mathcal{B}_1^*, \mathcal{F}_1]) + \operatorname{rank}(\Phi[\mathcal{F}_1, \cup_{i=2,3,4}\mathcal{F}_i]) + \operatorname{rank}(\Lambda[\cup_{i=2,3,4}\mathcal{B}_i^*, \cup_{i=2,3,4}\mathcal{F}_i]) - |\mathcal{F}_1| - 4 \\
=&|\mathcal{F}_1| - 1 + 4 + 4 - |\mathcal{F}_1| - 4 \\
=&3,
\end{aligned}
$$

which contradicts to equation (G.12). Thus, this case does not exist.

Next, we prove the second part. We denote by $\mathcal{B}_5^* = \{j_5, j_6\}$, $\mathcal{B}_6^* = \{j_7, j_8\}$ and $\mathcal{B}_7^* = \{j_9, j_{10}\}$. Since Λ, Φ and Ψ satisfy Condition 4, there exists three types of possible of bi-factor structure of $\mathcal{B}_5$, $\mathcal{B}_6$ and $\mathcal{B}_7$ and we discuss the three cases one by one. Without loss of generality, we assume D' given in the first part equals the identity matrix.

1. None of the bi-factor structures of the variables belonging to $\mathcal{B}_i^*$, $i = 5,6,7$, is correct. Without loss of generality, we assume $\mathcal{B}_5 = \{j_5, j_{10}\}$, $\mathcal{B}_6 = \{j_6, j_7\}$ and $\mathcal{B}_7 = \{j_8, j_9\}$. In this case, we consider the equation

$$\Sigma[\mathcal{B}_1^*, \mathcal{B}_5^*] = \Sigma^*[\mathcal{B}_1^*, \mathcal{B}_5^*],$$

which is equivalent to

$$
\begin{aligned}
\Lambda[\mathcal{B}_1^*, \{1\}]\lambda_{j_5,1} + \phi_{2,6}\Lambda[\mathcal{B}_1^*, \{2\}]\lambda_{j_5,6} &= \Lambda^*[\mathcal{B}_1^*, \{1\}]\lambda^*_{j_5,1} + \phi^*_{2,6}\Lambda^*[\mathcal{B}_1^*, \{2\}]\lambda^*_{j_5,6}, \\
\Lambda[\mathcal{B}_1^*, \{1\}]\lambda_{j_6,1} + \phi_{2,7}\Lambda[\mathcal{B}_1^*, \{2\}]\lambda_{j_6,7} &= \Lambda^*[\mathcal{B}_1^*, \{1\}]\lambda^*_{j_6,1} + \phi^*_{2,6}\Lambda^*[\mathcal{B}_1^*, \{2\}]\lambda^*_{j_6,6}.
\end{aligned}
$$

Since the first part is proved, we have assumed that $\Lambda[\mathcal{B}_1^*, \{1\}] = \Lambda^*[\mathcal{B}_1^*, \{1\}]$ and $\Lambda[\mathcal{B}_1^*, \{2\}] = \Lambda^*[\mathcal{B}_1^*, \{2\}]$. Noticing that $\Lambda^*[\mathcal{B}_1^*, \{1\}]$ and $\Lambda^*[\mathcal{B}_1^*, \{2\}]$ are linearly independent, we have $\lambda_{j_5,1} = \lambda^*_{j_5,1}$ and $\lambda_{j_6,1} = \lambda^*_{j_6,1}$. Similarly, by considering the equations $\Sigma[\mathcal{B}_1^*, \mathcal{B}_6^*] = \Sigma^*[\mathcal{B}_1^*, \mathcal{B}_6^*]$ and $\Sigma[\mathcal{B}_1^*, \mathcal{B}_7^*] = \Sigma^*[\mathcal{B}_1^*, \mathcal{B}_7^*]$, we have $\lambda_{j_7,1} = \lambda^*_{j_7,1}$, $\lambda_{j_8,1} = \lambda^*_{j_8,1}$, $\lambda_{j_9,1} = \lambda^*_{j_9,1}$ and $\lambda_{j_{10},1} = \lambda^*_{j_{10},1}$.

By considering the equation

$$\Sigma[j_5, j_6] = \Sigma^*[j_5, j_6],$$

which is equivalent to $\lambda_{j_5,1}\lambda_{j_6,1} + \phi_{6,7}\lambda_{j_5,6}\lambda_{j_6,7} = \lambda^*_{j_5,1}\lambda^*_{j_6,1} + \lambda^*_{j_5,6}\lambda^*_{j_6,6}$. We further have

$$\phi_{6,7}\lambda_{j_5,6}\lambda_{j_6,7} = \lambda^*_{j_5,6}\lambda^*_{j_6,6}. \tag{G.13}$$

We can similarly have the equations

$$\begin{aligned} \phi_{6,7}\lambda_{j_5,6}\lambda_{j_7,7} = & \quad \phi^*_{6,7}\lambda^*_{j_5,6}\lambda^*_{j_7,7}, \\ \phi_{6,7}\lambda_{j_{10},6}\lambda_{j_6,7} = & \quad \phi^*_{6,8}\lambda^*_{j_{10},8}\lambda^*_{j_6,6}, \\ \phi_{6,7}\lambda_{j_{10},6}\lambda_{j_7,8} = & \quad \phi^*_{7,8}\lambda^*_{j_{10},8}\lambda^*_{j_7,7}. \end{aligned} \tag{G.14}$$

By combining the equations (G.13) and (G.14), we have $\phi^*_{6,7}\phi^*_{6,8} = \phi^*_{7,8}$. In a symmetric manner, we further have $\phi^*_{6,8}\phi^*_{7,8} = \phi^*_{6,7}$ and $\phi^*_{6,7}\phi^*_{7,8} = \phi^*_{6,8}$. Since $\phi^*_{6,7}$, $\phi^*_{6,8}$ and $\phi^*_{7,8} \neq 0$, we have $|\phi^*_{6,7}\phi^*_{6,8}\phi^*_{7,8}| = 1$, which leads to $|\phi^*_{6,7}| = |\phi^*_{6,8}| = |\phi^*_{7,8}| = 1$ and violates the assumption that Φ^* is positive definite. Thus, this case does not exist.

2. Only one of the bi-factor structures of the variables belonging to $\mathcal{B}^*_i$, $i = 5, 6, 7$, is correct. Without loss of generality, we assume $\mathcal{B}_5 = \{j_5, j_6\}$, $\mathcal{B}_6 = \{j_7, j_9\}$ and $\mathcal{B}_7 = \{j_8, j_{10}\}$. By the same argument in the first case, we have $\lambda_{j_i,1} = \lambda^*_{j_i,1}$ for $i = 5, \ldots, 10$. Next, consider the equations on the diagonal entries of

$$\Sigma[\mathcal{B}^*_6 \cup \mathcal{B}^*_7, \mathcal{B}^*_6 \cup \mathcal{B}^*_7] = \Sigma^*[\mathcal{B}^*_6 \cup \mathcal{B}^*_7, \mathcal{B}^*_6 \cup \mathcal{B}^*_7].$$

we have the following 6 equations

$$\begin{aligned} &\phi_{7,8}\lambda_{j_7,7}\lambda_{j_8,8} = \lambda^*_{j_7,7}\lambda^*_{j_8,7}, \\ &\lambda_{j_7,7}\lambda_{j_9,7} = \phi^*_{7,8}\lambda^*_{j_7,7}\lambda^*_{j_9,8}, \\ &\phi_{7,8}\lambda_{j_7,7}\lambda_{j_{10},8} = \phi^*_{7,8}\lambda^*_{j_7,7}\lambda^*_{j_{10},8}, \\ &\phi_{7,8}\lambda_{j_8,8}\lambda_{j_9,7} = \phi^*_{7,8}\lambda^*_{j_8,7}\lambda^*_{j_9,8}, \\ &\lambda_{j_8,8}\lambda_{j_{10},8} = \phi^*_{7,8}\lambda^*_{j_8,7}\lambda^*_{j_{10},8}, \\ &\phi_{7,8}\lambda_{j_9,7}\lambda_{j_{10},8} = \lambda^*_{j_9,8}\lambda^*_{j_{10},8}. \end{aligned}$$

According to the first equation above, we have $\phi_{7,8} \neq 0$. By the 6 equations, we also have $(\phi^*_{7,8})^4\phi^4_{7,8} = \phi^2_{7,8}$. Then we have $|\phi^*_{7,8}| = |\phi_{7,8}| = 1$, which violates the assumption that Φ^* is positive definite. Thus, this case does not exist.

3. The bi-factor structure is correct. Without loss of generality, we assume $\mathcal{B}_5 = \{j_5, j_6\}$, $\mathcal{B}_6 = \{j_7, j_8\}$ and $\mathcal{B}_7 = \{j_9, j_{10}\}$. Similar to the previous argument, we first have $\lambda_{j_i,1} = \lambda^*_{j_i,1}$ for $i = 5, \ldots, 10$. Moreover, we have $\phi_{2,6}\lambda_{5,6} = \phi^*_{2,6}\lambda^*_{5,6}$, $\phi_{2,6}\lambda_{6,6} = \phi^*_{2,6}\lambda^*_{6,6}$ and $\lambda_{5,6}\lambda_{6,6} = \lambda^*_{5,6}\lambda^*_{6,6}$. These 3 equations leads to $\phi_{2,6} = \phi^*_{2,6}$, $\lambda_{5,6} = \lambda^*_{5,6}$ and $\lambda_{6,6} = \lambda^*_{6,6}$ or $\phi_{2,6} = -\phi^*_{2,6}$, $\lambda_{5,6} = -\lambda^*_{5,6}$ and $\lambda_{6,6} = -\lambda^*_{6,6}$. With the same argument, the loadings and correlations related with the variables belonging to $\mathcal{B}^*_6$ and $\mathcal{B}^*_7$

are also determined up to a sign flip. The check of $\psi_j = \psi_j^*$ for $j \in \mathcal{B}_i^*$, $i = 5, 6, 7$ are straight forward.

References

Alfonzetti, G., Bellio, R., Chen, Y., and Moustaki, I. (2024). Pairwise stochastic approximation for confirmatory factor analysis of categorical data. *British Journal of Mathematical and Statistical Psychology*.

Anderson, T. and Rubin, H. (1956). Statistical inference in factor analysis. In *Proceedings of the Berkeley Symposium on Mathematical Statistics and Probability*, page 111. University of California Press.

Fang, G., Guo, J., Xu, X., Ying, Z., and Zhang, S. (2021). Identifiability of bifactor models. *Statistica Sinica*, 31:2309–2330.

Jennrich, R. I. and Bentler, P. M. (2012). Exploratory bi-factor analysis: The oblique case. *Psychometrika*, 77(3):442–454.

Stan Development Team (2022). The stan core library. Version 2.33.

Yung, Y.-F., Thissen, D., and McLeod, L. D. (1999). On the relationship between the higher-order factor model and the hierarchical factor model. *Psychometrika*, 64:113–128.
